# Epidemiology, pathogen spectrum, and antimicrobial resistance of infections in burn patients stratified by total body surface area: a bicenter study with evaluation of targeted next-generation sequencing

**DOI:** 10.3389/fmicb.2026.1866146

**Published:** 2026-07-15

**Authors:** Zihao Ouyang, Yamin Lu, Lei Chen, Yanrong Lai, Pingsen Zhao

**Affiliations:** 1 Department of Laboratory Medicine, Yuebei People's Hospital Affiliated to Shantou University Medical College, Shaoguan, China; 2 Department of Clinical Laboratory, Guangzhou Red Cross Hospital of Jinan University, Guangzhou, China; 3 Laboratory for Diagnosis of Clinical Microbiology and Infection, Yuebei People's Hospital Affiliated to Shantou University Medical College, Shaoguan, China; 4 Research Center for Interdisciplinary & High-Quality Innovative Development in Laboratory Medicine, Shaoguan, China; 5 Shaoguan Municipal Quality Control Center for Laboratory Medicine, Yuebei People's Hospital Affiliated to Shantou University Medical College, Shaoguan, China; 6 Shaoguan Municipal Quality Control Center for Surveillance of Bacterial Resistance, Shaoguan, China; 7 Shaoguan Engineering Research Center for Research and Development of Molecular and Cellular Technology in Rapid Diagnosis of Infectious Diseases and Cancer, Shaoguan, China

**Keywords:** antimicrobial resistance, burn infection, pathogen spectrum, targeted next-generation sequencing, total body surface area

## Abstract

**Background:**

Infection remains a leading cause of morbidity and mortality in burn patients, particularly in those with extensive injuries. However, comprehensive analyses integrating pathogen distribution, antimicrobial resistance, and advanced molecular diagnostics across burn severity remain limited.

**Methods:**

We retrospectively analyzed 814 burn patients with infections admitted to two tertiary burn centers in southern China between 2020 and 2024, stratified by total body surface area (TBSA). Pathogen distribution, antimicrobial resistance patterns, complications, hospitalization costs, and length of stay were evaluated. In addition, 40 clinical specimens were prospectively analyzed using both conventional culture and targeted next-generation sequencing (t-NGS).

**Results:**

Among 814 infected burn patients stratified by TBSA (<10%, *n* = 358; 10–49%, *n* = 292; ≥50%, *n* = 164), polymicrobial infections rose from 25.7 to 64.4% (*p* < 0.001), Gram-negative bacteria from 27.1 to 56.6% (*p <* 0.001; e.g., *Acinetobacter baumannii* 14.2%, *Klebsiella pneumoniae* 18.9%), and fungi from 9.0 to 20.6% (*p* < 0.001; e.g., *Candida albicans* 5.2%). MDR organisms increased, with *A. baumannii* >85% resistant to cephalosporins/carbapenems. Severe burns were associated with higher complication rates (shock 70.7%, MODS 29.3%; *p* < 0.001), 58-day median LOS, and hospitalization costs of 554,246 CNY (*p* < 0.001). In the 40-specimen pilot diagnostic comparison, t-NGS showed 77.5% positivity and culture showed 55.0% positivity (*p* = 0.012), with a shorter turnaround time for t-NGS than culture (1.7 vs. 3.9 days; *p* < 0.001). In blood specimens, t-NGS positivity was 55.6% compared with 16.7% for conventional culture (*p* = 0.039).

**Conclusion:**

Burn severity is strongly associated with pathogen spectrum and antimicrobial resistance, underscoring the need for severity-stratified management strategies in burn care. In this pilot comparison, t-NGS showed a higher positivity rate than conventional culture and may serve as an adjunct diagnostic method. However, its clinical interpretation must be integrated with phenotypic data given the potential for colonization or genotypic-phenotypic discordance.

## 1 Introduction

Burns constitute a common yet highly complex form of trauma. Patients with severe burns are particularly predisposed to local wound or systemic infections resulting from skin barrier disruption, impaired immune function, and the frequent use of invasive procedures. Consequently, infection remains a primary complication and a leading driver of mortality in this population (Church et al., 2006). According to World Health Organization (WHO) data, approximately 11 million people sustain burns requiring medical intervention annually (Peck, 2011). Notably, 95% of burn-related fatalities occur in low- and middle-income countries. Although modern standard-of-care resuscitation protocols have significantly minimized early mortality from hypovolemic shock, infectious complications and subsequent sepsis remain the prominent threat, directly accounting for approximately 50 to 80% of late deaths in severely burned patients (Roy et al., 2024). The China Burn Prevention and Control Annual Report 2022 indicates that China experiences over 2 million burn cases each year, representing a 14.1% increase compared to 1990. Moderate-to-severe burns account for 18% of these cases, with infection incidence reaching as high as 43%. Furthermore, patients with third-degree burns exceeding 30% of the total body surface area (TBSA) face a sevenfold higher risk of infection than those with minor burns, significantly elevating mortality rates (Yang et al., 2024). Given the rising prevalence of antimicrobial-resistant strains, the prevention and management of burn infections have emerged as a critical priority in global clinical practice and public health.

In recent years, epidemiological and pathogen spectrum research on burn infections has expanded significantly both globally and within China. International multicenter studies indicate that Gram-negative bacteria—including *Pseudomonas aeruginosa*, *Acinetobacter baumannii*, and *Enterobacterales*—remain the predominant pathogens in burn wounds, accompanied by an escalating burden of antimicrobial resistance (Lachiewicz et al., 2017). While Chinese epidemiological patterns generally align with global trends, they exhibit distinct regional variations; for instance, the prevalence of fungi and specific opportunistic pathogens is notably higher in the humid, subtropical regions of southern China. Currently, conventional etiological methods, such as microbial culture, remain the cornerstone of clinical diagnosis. However, these techniques are often constrained by the complex microecology of burn wounds, prior antimicrobial administration, and the low abundance of certain pathogens, leading to suboptimal sensitivity and prolonged turnaround times. Such limitations impede the rapid and precise diagnostic capabilities essential for the effective management of burn infections.

Consequently, novel molecular diagnostic technologies—most notably targeted next-generation sequencing (t-NGS)—have garnered significant interest. By leveraging multiplex targeted amplification and deep sequencing of multiple pathogen-specific loci, t-NGS enables the simultaneous detection of bacteria, fungi, and key antimicrobial resistance (AMR) genes. This methodology offers accelerated turnaround times, markedly enhanced sensitivity for low-abundance pathogens, and reduced interference from commensal or environmental background microbiota (Huang et al., 2026). While previous studies have validated the diagnostic utility of t-NGS in severe, refractory, and culture-negative infections (Long et al., 2024), systematic research regarding its clinical application in burn care remains limited. Specifically, its performance across diverse infection phenotypes, hospitalization phases, and varying antibiotic exposure conditions warrants further investigation (Wu and Huang, 2021).

## 2 Materials and methods

### 2.1 Source of medical records

This bicenter study, integrating a retrospective cohort analysis with a prospective diagnostic comparison, was conducted across two tertiary burn centers in Southern China—Guangzhou Red Cross Hospital and Yuebei People’s Hospital Affiliated to Shantou University Medical College, from January 2020 to December 2025.

For the retrospective component of this bicenter study, 814 infected burn patients admitted to the Burn Departments of Guangzhou Red Cross Hospital and Yuebei People’s Hospital between January 2020 and December 2024 were enrolled. Participants were stratified by TBSA into three categories: mild (<10%), moderate (10–49%), and severe (≥50%). Furthermore, in a prospective arm spanning July 2024 to December 2025, 40 clinical specimens were collected from 29 patients at Guangzhou Red Cross Hospital for paired comparison using conventional culture and t-NGS. Specimens in the prospective cohort were enrolled non-consecutively using an event-triggered, indicator-driven protocol rather than routine convenience sampling. To ensure high-level clinical oversight, the determination of clinical deterioration nodes and subsequent sample collection were strictly evaluated and performed by experienced burn specialists (at the rank of attending physician or above). Crucially, to minimize the confounding effect of active antimicrobial suppression on conventional cultures, specimens were prioritized for collection either prior to the initiation of any new empirical antibiotic regimen or immediately before the next scheduled antimicrobial dose (at the trough concentration).

Sampling was immediately triggered when a patient met any of the following standardized clinical criteria suggestive of a suspected invasive infection with hyperacute onset:

1. Sudden systemic febrile response, defined as an acute onset of high fever (> 39 °C) or unexpected hypothermia (<36.5 °C), accompanied by clinical chills or unexplained fluctuations in pulse oxygen saturation (SpO_2_).
2. Acute deterioration of local wounds, such as unexplained necrosis or sloughing of skin grafts or flaps, accelerated sloughing or premature separation of burn eschar, or sudden spreading erythema and edema with purulent exudate around wound margins.
3. Abrupt respiratory decompensation, defined as an unexplained, progressive drop in the oxygenation index (PaO₂/FiO₂), or a change in the gross appearance of airway secretions to thick, purulent consistency.
4. A sharp surge in systemic inflammatory biomarkers, characterized by an unexpected doubling or marked elevation of white blood cell (WBC) count, procalcitonin (PCT), or C-reactive protein (CRP) within 24 h.

*Inclusion criteria*: (1) Patients admitted to the Burn Departments of Guangzhou Red Cross Hospital or Yuebei People’s Hospital between January 2020 and December 2024, including those discharged upon recovery or deceased during hospitalization. (2) A primary diagnosis of burns, including thermal burns (flame or scald), chemical burns, electrical injuries, thermal crush injuries, or cold injuries. (3) Confirmed clinical and microbiological evidence of infection during the hospital stay, satisfying the predefined diagnostic criteria that strictly distinguish true infection from colonization. (4) Availability of comprehensive and complete medical records.

*Exclusion criteria*: (1) Patients for whom burns were not the primary reason for admission or the initial diagnosis. (2) Patients with historical burn injuries admitted specifically for secondary infections of chronic wounds or elective scar-related surgeries. (3) Patients with incomplete or inaccessible clinical data.

Interpretation of Polymicrobial Growth and Handling of Repeated Isolates: (1) Polymicrobial isolates: Mixed microbial growth detected within a single specimen was evaluated using stringent diagnostic criteria. Cases with polymicrobial growth in the absence of local invasive necrosis or clinical manifestations consistent with systemic inflammatory response syndrome (SIRS) were classified as polymicrobial colonization. Only cases confirmed as infection based on electronic medical record review were defined as true polymicrobial infection. (2) Duplicate Isolates: To prevent the artificial inflation of pathogen frequencies and antimicrobial resistance (AMR) rates due to prolonged hospital stays and repeated sampling, duplicate isolates were stringently filtered. Only the first unique isolate of a specific pathogen species per patient per infection episode (within a 14-day window) was included in the final epidemiological and resistance profiling analysis. Subsequent identical isolates from the same patient and site were treated as repeated surveillance cultures and excluded.

### 2.2 t-NGS methods and materials

Specimens were processed at the Clinical Precision Testing Center of Guangzhou Red Cross Hospital using a t-NGS workflow. This involved multiplex targeted amplification followed by high-throughput sequencing, with sequences aligned against the NCBI Genomes database^1^ for pathogen identification.

The technical pipeline comprised the following stages: (1) Nucleic Acid Extraction: Microbial DNA was isolated from clinical specimens using the QIAamp DNA Mini Kit (Qiagen, Germany) via automated or manual protocols according to the manufacturer’s instructions. When host-background reduction was applied, it was performed as a selective pre-lysis depletion step before microbial cell disruption. Briefly, extracellular and host-derived DNA was digested by DNase while microbial cells remained structurally intact, thereby minimizing degradation of microbial genomic DNA. The DNase was subsequently inactivated or removed before microbial lysis and DNA purification. This workflow was used only where appropriate according to the validated laboratory protocol. RNA viruses were not included in the analytical scope of this study; therefore, no RNA extraction or reverse-transcription step was performed. (2) Library Preparation and Panel Composition: Targeted amplification was performed using a comprehensive, ultra-multiplex PCR panel designed to simultaneously enrich bacterial, fungal, selected DNA viral, and major antimicrobial resistance (AMR) gene targets. Specifically, the panel contained highly optimized primer sets targeting the 16S rRNA gene for bacteria, the internal transcribed spacer (ITS1/ITS2) regions for fungi, conserved genomic regions of selected clinically relevant DNA viruses, and key AMR determinants—including but not limited to *mecA, bla_OXA-23, bla_OXA-48, bla_KPC*, and *vanA*. Multiplex PCR amplicons were subsequently purified, and sequencing adapters along with unique sample barcodes were ligated to the fragments to facilitate sample multiplexing and downstream high-throughput sequencing. (3) Nanopore Sequencing: Prepared libraries were loaded onto a MinION Mk1D flow cell (Oxford Nanopore Technologies, UK) and sequenced in real time. Data acquisition was continued until each sample achieved a minimum sequencing depth of 100,000 reads. (4) Bioinformatics Pipeline: Real-time basecalling of raw electrical signals was performed using Guppy, which was integrated into the MinKNOW platform. Raw reads were filtered to obtain clean data by removing sequences with an average Phred quality score (Q-score) < 10 or read length <100 bp. The qualified clean reads were then subjected to homology search and functional annotation against public reference databases using BLAST. (5) Bioinformatic Thresholds and Clinical Interpretation Rules: To minimize false positives resulting from environmental or reagent cross-contamination, a no-template control (NTC) was processed and sequenced in parallel with each batch of clinical specimens. Stringent reporting thresholds and read-count criteria were established as follows:

- Bacteria and Fungi: A microbial species was considered positive if it yielded ≥10 specific mapping reads, and the read count was at least 10-fold higher than that detected in the concurrent NTC.
- Viruses: Given the smaller genome sizes of viruses and their clinical relevance in immunocompromised burn patients, viral detection was defined as positive when ≥3 specific reads mapped to the reference viral genome.
- Antimicrobial Resistance (AMR) Genes: An AMR gene was reported as positive if it met a minimum threshold of ≥10 specific reads and achieved a target gene sequence coverage of ≥50%; the genotypic resistance profile was interpreted in strict correlation with the taxonomic identification of its potential host bacteria.

To be considered a true pathogen rather than colonization or contamination, the t-NGS positive microorganism had to strictly align with localized inflammatory tissue reactions, systemic inflammatory response syndrome (SIRS), or progressive radiographic findings according to the American Burn Association consensus definitions (The American Burn Association Consensus Conference on Burn Sepsis and Infection Group et al., 2007).

### 2.3 Conventional culture methods

The technical pipeline comprised the following stages: (1) Microbial culture: Blood culture samples comprised one aerobic and one anaerobic bottle per set, with two sets collected from distinct puncture sites. Bottles were incubated at approximately 37 °C for up to 5 days using the BD BACTEC™ FX semiautomated continuous monitoring system (Becton, Dickinson, and Company, USA). Positive cultures underwent Gram staining and subculturing on solid media. All non-blood clinical specimens were collected under strict aseptic conditions and transported to the microbiology laboratory within 2 h of collection. Wound secretions were collected using sterile swabs from deep wound beds after removing superficial debris, and immediately inoculated onto blood agar plates and Sabouraud dextrose agar plates (Guangdong Jiangmen Kailin Trading Co., Ltd.). Sputum and respiratory samples (including bronchoalveolar lavage fluid) were obtained via deep expectoration or endotracheal aspiration, and then seeded onto blood agar and chocolate agar plates to isolate routine and fastidious respiratory pathogens. Catheter tips (~5 cm) were processed and cultured using the Maki roll-plate semi-quantitative method. Mid-stream urine was collected into sterile containers and thoroughly mixed; a 1-μL calibrated loop was applied for standardized quantitative tree-streak culture on blood agar plates, while residual urine was centrifuged at 3,000 rpm for 8 min for fungal screening, with the concentrated sediment inoculated onto Sabouraud dextrose agar plates after discarding the supernatant. Tissue biopsies were minced and homogenized in sterile physiological saline under aseptic conditions before being inoculated onto blood agar and Sabouraud dextrose agar plates. The microbiological interpretation and clinical adjudication of these cultures (distinguishing true infection from colonization) strictly followed the pre-defined diagnostic consensus outlined below. (2) Microbial identification: MacConkey agar or other selective/differential media were not utilized during the initial culture phase. Instead, suspected single colonies were directly selected from the primary agar plates and identified via matrix-assisted laser desorption/ionization time-of-flight mass spectrometry (MALDI-TOF-MS) using bioMérieux instruments (bioMérieux, France) to achieve precise identification of Gram-negative pathogens. (3) Antimicrobial Susceptibility Testing: Susceptibility testing employed the VITEK® 2 COMPACT (bioMérieux, France) and BD Phoenix™ (Becton, Dickinson and Company, USA) automated systems. For the retrospective cohort (2020–2024), bacterial antimicrobial susceptibility testing (AST) results were interpreted strictly according to the contemporaneous annual updates of the Clinical and Laboratory Standards Institute (CLSI) M100 guidelines relevant to each respective year (ranging from the 30th edition in 2020 to the 34th edition in 2024) to accurately reflect real-time clinical definitions. Major breakpoint updates during this study period—specifically the 2022 revision of piperacillin-tazobactam breakpoints for Enterobacterales and the 2023 revision for *Pseudomonas aeruginosa*—were dynamically accounted for via year-specific laboratory expert system rules. Because the operational definition of Multidrug-Resistant (MDR) status relies on non-susceptibility (including Intermediate, SDD, or Resistant phenotypes), a sensitivity re-analysis confirmed that these historical breakpoint shifts exerted no material impact on the core results, epidemiological resistance trends, or statistical conclusions of this study. For antifungal susceptibility testing (AFST), minimum inhibitory concentrations (MICs) obtained via the automated VITEK-2 system were evaluated by concurrently referencing both the CLSI (CLSI M27M44) guidelines and the European Committee on Antimicrobial Susceptibility Testing (EUCAST) breakpoint tables appropriate for each historical year.

### 2.4 Definitions and genotype–phenotype comparison of critical resistance phenotypes

Critical multidrug-resistant and carbapenem-resistant phenotypes were operationally categorized as follows: (1) Multidrug-Resistant (MDR): Defined as acquired non-susceptibility to at least one agent in three or more antimicrobial categories clinically approved for the specific bacterial genus, based on international standard consensus definitions. (2) Carbapenem-Resistant *Acinetobacter baumannii* (CRAB) and *Pseudomonas aeruginosa* (CRPA): Defined as *A. baumannii complex* or *P. aeruginosa* isolates demonstrating clinical resistance to imipenem or meropenem according to the applicable annual criteria. (3) Carbapenem-Resistant *Enterobacterales* (CRE): Defined as any *Enterobacterales* isolate displaying non-susceptibility or resistance to at least one carbapenem (imipenem, meropenem, doripenem, or ertapenem) as defined by the concurrent CLSI guidelines. Phenotypic indications were cross-verified via the expert system software of automated instruments. (4) MRSA and MRSE: Methicillin-Resistant *Staphylococcus aureus* (MRSA) and Methicillin-Resistant *Staphylococcus epidermidis* (MRSE) were screened and determined using the cefoxitin disk/well test as a surrogate marker for oxacillin resistance per annual guidelines. (5) Genotype–Phenotype Comparison: For the critical resistance phenotypes identified in the prospective cohort (CRE, CRAB, CRPA, and MRSA/MRSE), phenotypic AST profiles were compared with corresponding key resistance determinants detected by t-NGS, including *mecA* for MRSA/MRSE, *bla_OXA-23* for CRAB, and *bla_KPC*, *bla_NDM*, or *bla_OXA-48*-like for CRE, where applicable.

### 2.5 Clinical and microbiological definitions of infection

To minimize selection bias and avoid confounding from bacterial colonization, contamination, or biofilm carriage, all included cases were cross-evaluated using clinical, laboratory, and microbiological data. The diagnostic criteria were based on the American Burn Association (ABA) consensus definitions and the Centers for Disease Control and Prevention/National Healthcare Safety Network (CDC/NHSN) guidelines adapted for burn injuries: (1) Burn Wound Infection (BWI): Diagnosed by local wound manifestations (e.g., unexpected eschar separation, dark brown/black discoloration of the wound, rapid extension of erythema or edema at the wound margins, or purulent drainage) accompanied by positive qualitative or semi-quantitative cultures from wound secretions or tissue biopsies. Simple microbial isolation without local inflammatory tissue reaction or systemic symptoms was classified as colonization and excluded. (2) Bloodstream Infection (BSI): Defined as at least one positive blood culture for a recognized pathogen paired with systemic symptoms (e.g., fever >38 °C or hypothermia <36 °C, tachycardia, or leukocytosis). For common skin commensals (e.g., coagulase-negative staphylococci), two separate positive blood cultures drawn on different occasions were required to rule out contamination. (3) Pneumonia: Diagnosed based on new or progressive and persistent infiltrates on chest radiography, combined with at least two clinical signs (fever, purulent sputum, or leukocytosis/leukopenia) and microbiological confirmation via culture of sputum, endotracheal aspirates, or bronchoalveolar lavage fluid (BALF). (4) Catheter-Related Bloodstream Infection (CRBSI): Defined as a positive semi-quantitative culture (≥15 CFU per catheter segment) or quantitative culture (≥10^3^ CFU/mL) of the catheter tip, showing the same organism as a concurrent peripheral blood culture, with localized or systemic signs of infection. (5) Urinary Tract Infection (UTI): Diagnosed by a quantitative urine culture showing ≥10^5^ CFU/mL of no more than two species of microorganisms, accompanied by relevant clinical symptoms (e.g., fever, costovertebral angle tenderness) or iatrogenic parameters in catheterized patients.

### 2.6 Ethics approval and consent to participate

The study was approved by Clinical Research Ethics Committee of Yuebei People’s Hospital Affiliated to Shantou University Medical College and Guangzhou Red Cross Hospital of Jinan University.

### 2.7 Statistical analysis

Statistical analyses were performed using GraphPad Prism (version 9.5.0; GraphPad Software Inc., San Diego, CA, USA) and SPSS (version 26.0; IBM Corp., Armonk, NY, USA). Data management and initial processing were conducted using WPS Spreadsheets (Kingsoft Office, Beijing, China). Continuous variables were presented as mean ± standard deviation (SD) for normally distributed data or as median (interquartile range, IQR) for non-normally distributed data. Categorical variables were expressed as frequencies and percentages. Comparisons of independent categorical variables were performed using the chi-square test, while paired categorical data were analyzed with McNemar’s test. Cohen’s kappa coefficient was calculated to assess the consistency between the two methods. For continuous variables, independent samples *t*-tests were used for normally distributed data, whereas the Mann–Whitney *U* test was applied for independent non-normally distributed variables. For paired non-normally distributed continuous variables, the Wilcoxon signed-rank test was employed. We performed multivariable linear regression to identify independent factors associated with hospitalization costs and length of stay (LOS). Given the inherent right-skewness of the data, both hospitalization costs and LOS were natural log-transformed prior to modeling to satisfy the normality and homoscedasticity assumptions of ordinary least squares (OLS) regression. A total of 10 variables were included in the hospitalization-cost model and 9 variables in the LOS model. Candidate covariates included TBSA, inhalation injury, number of surgical procedures, tracheal intubation, smoking status, initial microbe detection status, and other clinically relevant variables. All covariates were selected based on clinical experience and relevance and were simultaneously entered into the models using the forced entry (“Enter”) method. The variance inflation factor (VIF) was used to assess multicollinearity among the variables, with a VIF < 5 considered acceptable to ensure stable regression coefficients. A two-sided *p* < 0.05 was defined as statistically significant.

## 3 Results

### 3.1 Patient demographics

**Figure 1:**
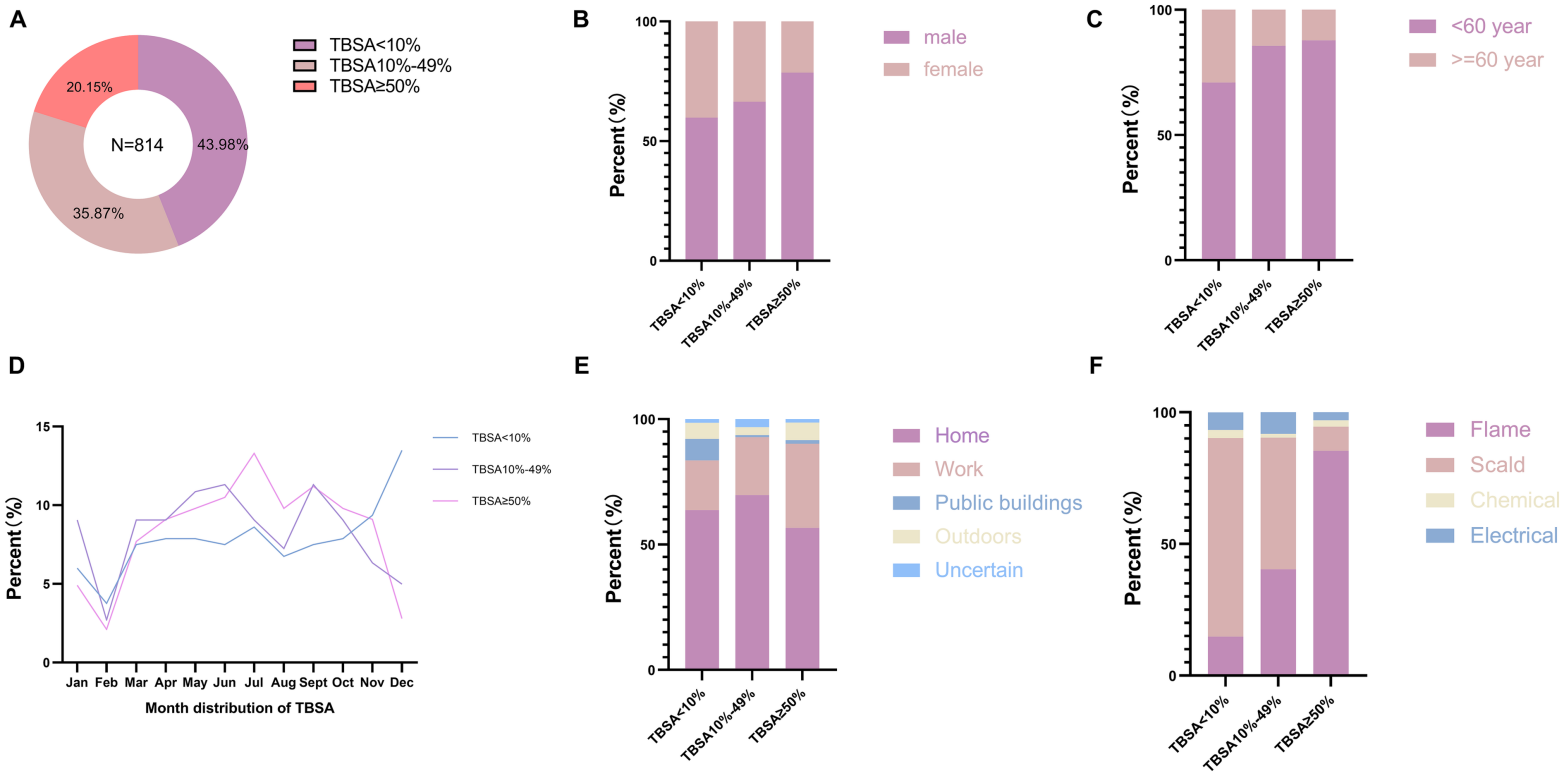
Demographic and clinical characteristics of burn patients stratified by TBSA. **(A)** Overall patient distribution (<10%, 10–49%, and ≥50% TBSA). **(B)** Gender distribution. **(C)** Age group distribution. **(D)** Monthly case distribution. **(E)** Injury locations. **(F)** Burn etiologies. Data are from 814 patients across two tertiary burn centers; categorical variables are shown as percentages.

Figure 1 presents the overall demographic and clinical characteristics of the 814 infected burn patients included in this study. Patients with mild burns (TBSA < 10%) constituted the largest cohort (358/814, 43.98%), whereas those with severe burns (TBSA ≥ 50%) represented the smallest proportion (164/814, 20.15%; Figure 1A). Regarding gender distribution, males significantly outnumbered females; however, this gender disparity was less pronounced in the mild burn group compared to the moderate and severe cohorts (Figure 1B). The majority of patients were young or middle-aged adults under 60 years (Figure 1C). Seasonal analysis of the infected cohort indicated that the monthly distribution of moderate and severe burn infection cases was descriptively higher in June, July, and September, with the highest number of cases observed in June. In contrast, the number of infected mild burn cases showed a relative peak in December, coinciding with fewer concurrent moderate and severe cases during this fmonth (Figure 1D). The home was the predominant location of injury, followed by the workplace (Figure 1E). The primary etiologies were flame burns and scalds. Stratification by severity revealed that mild-to-moderate burns were predominantly caused by scalds, whereas severe burns were primarily attributable to flame injuries (Figure 1F). The overall in-hospital mortality among enrolled patients was 1.47% (12/814). Notably, mortality rose in a distinct stepwise manner as TBSA increased, from 0% in the mild TBSA group to 6.71% (11/164) in the severe group (Supplementary Table 2).

Further analysis of burn etiology stratified by age and gender revealed distinct age-specific clustering. The 20–44-year age group exhibited the highest incidence of flame, chemical, and electrical burns among infected patients. Scalds were the predominant etiology in young children (0–4 years) and older adults (≥60 years), while the overall incidence of all burn types was lowest in the 5–19-year age group (Supplementary Figure 1A). Regarding gender, males predominated across all etiological categories. Notably, chemical and electrical burns were observed almost exclusively in males, whereas the gender distribution for scalds remained relatively balanced (Supplementary Figure 1B).

### 3.2 The frequency of major complications increased sharply with increasing TBSA among infected burn patients

An anatomical analysis of burn sites stratified by severity revealed distinct distribution patterns. Patients with mild burns primarily presented with injuries to the lower (195/358, 54.47%) and upper (121/358, 33.80%) extremities. In contrast, patients with moderate-to-severe burns exhibited a higher propensity for multi-site involvement, while the perineal region remained the least affected site overall (Supplementary Figure 2). Supplementary Table 1 details the frequency of complications among infected burn patients stratified by TBSA groups (<10, 10–49%, and ≥50%). Generally, the frequency of complications increased with TBSA among infected burn patients. Severe complications, such as inhalation injury and shock, were rare in the mild group (TBSA < 10%; e.g., inhalation injury 12/358, 3.35%, shock 3/358, 0.84%) but markedly elevated in the severe group (TBSA ≥ 50%; inhalation injury 126/164, 76.83%, shock 116/164 70.73%; *p* < 0.001). Notably, life-threatening conditions including multiple organ dysfunction syndrome (MODS) and acute respiratory distress syndrome (ARDS) were absent in the mild group but affected 29.27% (48/164) and 17.07% (28/164) of the severe group, respectively (*p* < 0.001). Statistically significant inter-group differences were also observed for wound infection, pneumonia, and systemic inflammatory response syndrome (SIRS; all *p* ≤ 0.001), with pneumonia peaking at 17.68% (29/164) in the severe group cohort, which was accompanied by a significantly higher rate of tracheal intubation in the severe group compared to the mild burn group (Supplementary Table 2). Conversely, urinary tract infections (UTI) showed no significant association with burn severity (*p* = 0.47), maintaining a consistently low incidence (2.23–3.05%) across all groups.

### 3.3 Hospitalization costs and length of stay increased progressively with burn severity

Hospitalization costs and length of stay (LOS) demonstrated a progressive increase corresponding to burn severity (Supplementary Figure 3). The median cost and LOS were 23,927 CNY (1 USD ≈ 7.2 CNY) and 19 days for mild burns, rising to 92,291 CNY and 26 days for moderate burns, and peaking at 554,245.5 CNY and 58 days for severe burns. To further identify the independent predictors of hospitalization costs and LOS, multiple linear regression analyses were performed on the log-transformed variables ln(cost) and ln(LOS). Regarding hospitalization costs (Table 1), the number of surgeries (unstandardized *β* = 0.300, 95% CI: 0.246 to 0.354, *p* < 0.001) and TBSA (unstandardized *β* = 0.016, 95% CI: 0.012 to 0.019, *p* < 0.001) were identified as strong, independent positive predictors, with the number of surgeries exerting the greatest relative impact (standardized *β* = 0.455). Inhalation injury also significantly contributed to higher costs (unstandardized *β* = 0.354, 95% CI: 0.153 to 0.555, *p* < 0.001). Conversely, a positive microbe detection at first admission was associated with a significant decrease in costs (unstandardized *β* = −0.223, 95% CI: −0.372 to −0.074, *p* = 0.003). Other factors, including LOS, smoking status, tracheal intubation, treatment outcome, cause, and location of burn, showed no statistically significant associations with hospitalization costs (all *p* > 0.05). For the length of stay (Table 2), the number of surgeries remained the most dominant positive factor (standardized *β* = 0.678, unstandardized *β* = 0.239, 95% CI: 0.214 to 0.263, *p* < 0.001). Additionally, the cause of burn (unstandardized *β* = 0.056, 95% CI: 0.003 to 0.110, *p* = 0.039) and location of burn (unstandardized *β* = 0.042, 95% CI: 0.003 to 0.082, *p* = 0.035) were positively correlated with longer LOS. In contrast, better treatment outcome (unstandardized *β* = −0.209, 95% CI: −0.285 to −0.134, *p* < 0.001) and positive microbe detection at first admission (unstandardized *β* = −0.140, 95% CI: −0.225 to −0.054, *p* < 0.001) were significant independent factors mitigating LOS. Inhalation injury, smoking status, TBSA, and tracheal intubation did not significantly influence LOS (all *p* > 0.05).

**Table 1:** Multivariable linear regression analysis of factors associated with log-transformed hospitalization costs.

Independent variables	Unstandardized *β* coefficients	Standardized *β* coefficients	*t*	*p* value	95% CI	
					Lower	Upper
Inhalation injury	0.354	0.105	3.464	<0.001	0.153	0.555
First admission was microbe detection positive	−0.223	−0.074	−2.943	0.003	−0.372	−0.074
Number of surgeries	0.3	0.455	10.884	<0.001	0.246	0.354
length of stay (LOS)	0.003	0.057	1.621	0.105	−0.001	0.006
Smoking status	0.274	0.04	1.85	0.065	−0.017	0.565
TBSA	0.016	0.298	8.136	<0.001	0.012	0.019
Tracheal intubation	0.019	0.006	0.212	0.832	−0.161	0.2
Treatment outcome	−0.029	−0.01	−0.431	0.667	−0.161	0.103
Cause of burn	0.027	0.014	0.57	0.569	−0.066	0.121
Location of burn	0.044	0.028	1.271	0.204	−0.024	0.113

Hospitalization costs were log-transformed (ln-transformed) prior to regression analysis to satisfy the assumption of normality for residuals. A total of 10 independent variables were included in the analysis, including TBSA, number of surgical procedures, inhalation injury, and clinically relevant variables. Results are presented as unstandardized *β* coefficients, standardized *β* coefficients, *t* values, *p* values, and 95% confidence intervals (CI).

**Table 2:** Multivariable linear regression analysis of factors associated with log-transformed length of stay (LOS).

Independent variables	Unstandardized *β* coefficients	Standardized *β* coefficients	*t*	*p* value	95% CI	
					Lower	Upper
Inhalation injury	0.105	0.058	1.791	0.074	−0.01	0.22
First admission was microbe detection positive	−0.14	−0.086	−3.212	<0.001	−0.225	−0.054
Number of surgeries	0.239	0.678	19.259	<0.001	0.214	0.263
Smoking status	0.134	0.037	1.611	0.108	−0.029	0.297
TBSA	0.002	0.061	1.563	0.118	0	0.004
Tracheal intubation	0.085	0.048	1.616	0.107	−0.018	0.187
Treatment outcome	−0.209	−0.131	−5.431	<0.001	−0.285	−0.134
Cause of burn	0.056	0.056	2.064	0.039	0.003	0.11
Location of burn	0.042	0.05	2.112	0.035	0.003	0.082

Hospitalization length of stay (LOS) were log-transformed (ln-transformed) prior to regression analysis to satisfy the assumption of normality for residuals. A total of 9 independent variables were included in the analysis, including TBSA, number of surgical procedures, inhalation injury, and clinically relevant variables. Results are presented as unstandardized *β* coefficients, standardized *β* coefficients, *t* values, *p* values, and 95% confidence intervals (CI).

### 3.4 Gram-positive bacteria decreased whereas gram-negative bacteria increased with TBSA

**Figure 2:**
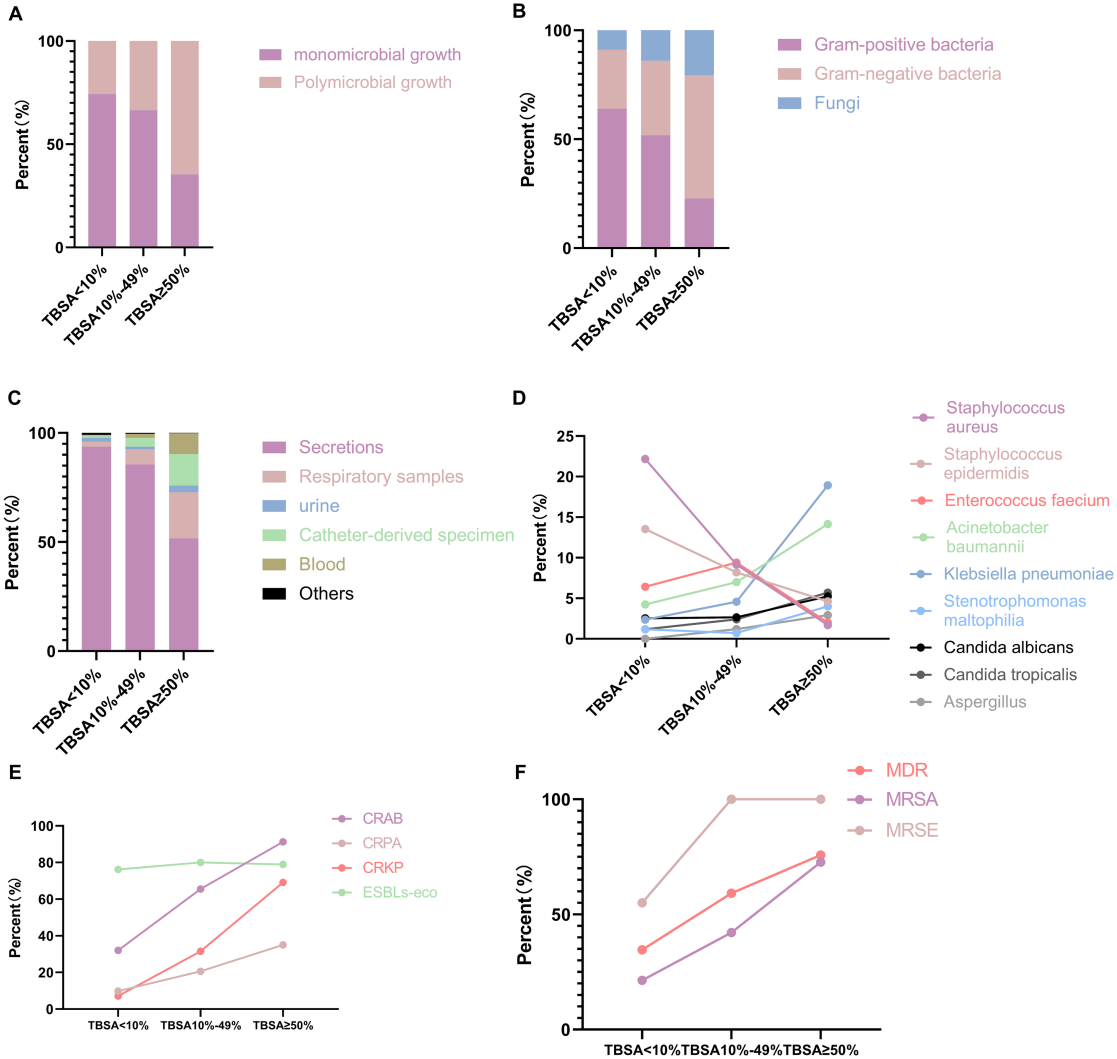
Microbiological characteristics of burn wound infections stratified by TBSA. **(A)** Monomicrobial and polymicrobial infection distribution. **(B)** Composition of major microbial categories (Gram-positive, Gram-negative, and fungi). **(C)** Specimen source distribution (respiratory samples include sputum, endotracheal aspirates, and bronchoalveolar lavage fluid). **(D)** Predominant bacterial species. **(E)** Antimicrobial resistance patterns of Gram-negative isolates. **(F)** Resistance patterns of total MDR and Gram-positive isolates. Data are derived from 1,656 culture isolates; percentages are compared using the chi-square test.

As illustrated in Figure 2, distinct variations in etiological patterns and microbial composition were observed across groups stratified by TBSA. Monomicrobial infections predominated in the mild and moderate burn groups (266/358, 74.3% and 194/292, 66.44%). However, increasing burn severity was associated with a marked shift toward polymicrobial infections, the prevalence of which rose to 64.43% (106/164) in the severe group (Figure 2A).

Analysis of specimen distribution identified wound secretions as the predominant source, followed by respiratory samples, urine, and catheter-related samples. While the overall proportion of positive blood cultures was relatively low, patients with extensive burns (TBSA ≥ 50%) exhibited a more diverse specimen profile. In this high-severity cohort, the dominance of secretion specimens diminished (336/650, 51.69%), accompanied by notable increases in the proportions of respiratory samples (137/650, 21.08%), catheter-tips (94/650, 14.46%), and blood (62/650, 9.54%; Figure 2C).

Regarding microbial composition, Gram-positive bacteria predominated in the mild burn group (TBSA < 10%), accounting for 63.96% (378/591) of isolates. However, this prevalence exhibited a significant inverse correlation with burn severity, declining to 51.81% (215/415) in the moderate group and 22.77% (148/650) in the severe group (TBSA ≥ 50%; *p* < 0.001). Conversely, the detection rate of Gram-negative bacteria demonstrated a significant positive association with TBSA, rising from 27.07% (160/591) in mild burns to 34.22% (142/415) and 56.62% (368/650) in the moderate and severe groups, respectively (*p* < 0.001; Figure 2B).

Further analysis of specific pathogen distribution (Supplementary Table 3) revealed distinct trends stratified by burn severity. Among Gram-positive bacteria, the prevalence of *Staphylococcus aureus* exhibited a significant inverse correlation with TBSA, decreasing from 22.17% (131/591) in the mild group (TBSA < 10%) to 1.69% (11/650) in the severe group (TBSA ≥ 50%; *p* < 0.001). *Staphylococcus epidermidis* followed a similar declining trajectory (13.54% vs. 4.62%; *p* < 0.001). *Enterococcus faecium* detection peaked in the moderate group (TBSA 10–49%) at 9.40% (39/415) but declined significantly to 2.00% (13/650) in the severe group (*p* < 0.001). No statistically significant differences were observed for other Gram-positive species.

Conversely, key Gram-negative pathogens demonstrated a strong positive association with burn severity. The detection rates of *Acinetobacter baumannii* and *Klebsiella pneumoniae* rose significantly with increasing TBSA, reaching 14.15% (92/650) and 18.92% (123/650), respectively, in the severe cohort (both *p* < 0.001). Similarly, *Stenotrophomonas maltophilia* was significantly enriched in the TBSA ≥ 50% group compared to other strata (26/650, 4.00%; *p* < 0.001). *Escherichia coli* showed a divergent pattern, with prevalence peaking in the moderate group (25/415, 6.02%) before decreasing in the severe group (19/650, 2.92%; *p* = 0.04). Fungal detection rates increased progressively with TBSA, rising from 8.97% (53/591, mild) to 13.98% (58/415, moderate) and 20.62% (134/650, severe; *p* < 0.001). Specifically, *Candida albicans*, *Candida tropicalis*, and *Aspergillus* spp. were notably more prevalent in patients with extensive burns (TBSA ≥ 50%; Figure 2D).

### 3.5 Multidrug resistance among gram-negative bacteria increased with burn severity

Supplementary Table 4 details the antimicrobial resistance profiles of predominant Gram-negative bacteria stratified by TBSA groups (<10%, 10–49%, and ≥50%). Generally, resistance rates to most antimicrobial agents demonstrated a positive correlation with burn severity, culminating in a marked surge in multidrug-resistant (MDR) isolates within the severe cohort (TBSA ≥ 50%; Figure 2F). Specifically, *Acinetobacter baumannii* in the ≥50% TBSA group exhibited resistance rates exceeding 85% for third- and fourth-generation cephalosporins, *β*-lactam/*β*-lactamase inhibitor combinations, and carbapenems. In contrast, isolates from lower TBSA groups were less resistant, and polymyxins remained highly active across all strata (Figure 2E). While *Pseudomonas aeruginosa* displayed lower overall resistance compared to *A. baumannii*, the ≥50% TBSA group showed significantly elevated resistance to ceftazidime, piperacillin-tazobactam, and carbapenems, although susceptibility to amikacin and polymyxins was largely preserved. For *Klebsiella pneumoniae*, the severe burn group exhibited resistance rates surpassing 70% for cephalosporins, fluoroquinolones, and trimethoprim-sulfamethoxazole. Similarly, *Escherichia coli* demonstrated an escalating resistance trend corresponding to TBSA, particularly against fluoroquinolones and trimethoprim-sulfamethoxazole, whereas resistance to aminoglycosides and carbapenems remained relatively low.

Supplementary Table 5 details the antimicrobial resistance profiles of major Gram-positive bacteria, stratified by TBSA. Overall, resistance rates for *Staphylococcus aureus* and *Staphylococcus epidermidis* demonstrated a positive correlation with burn severity, characterized by a marked escalation in multidrug-resistant (MDR) phenotypes (Figure 2F). In contrast, the resistance profiles of *enterococci* remained relatively stable. For *S. aureus*, penicillin resistance was ubiquitous across all cohorts, whereas resistance to oxacillin, clindamycin, erythromycin, fluoroquinolones, and tetracyclines increased significantly with TBSA, peaking in the ≥50% group. Notably, no resistance to vancomycin, linezolid, or teicoplanin was detected. *S. epidermidis* exhibited substantial resistance to penicillin and oxacillin, reaching 100% in the ≥50% TBSA group. Resistance to trimethoprim-sulfamethoxazole, aminoglycosides, and fluoroquinolones also increased markedly in moderate-to-severe burns, while susceptibility to glycopeptides and linezolid was fully preserved. Regarding *Enterococcus faecium*, isolates remained universally susceptible to vancomycin, linezolid, and teicoplanin. However, resistance to erythromycin and tetracyclines was elevated, particularly in the ≥50% TBSA group, while fluoroquinolone resistance remained comparatively low.

As detailed in Supplementary Table 6, *Candida albicans* generally maintained high susceptibility to voriconazole and fluconazole, with no resistant isolates detected in the severe burn group (TBSA ≥ 50%). In stark contrast, *Candida tropicalis* exhibited substantially elevated resistance to both azoles, demonstrating a positive correlation with increasing burn surface area. Specifically, resistance rates for voriconazole and fluconazole in the TBSA ≥ 50% cohort peaked at 48.28%, markedly surpassing the rates observed in the mild and moderate groups.

### 3.6 Pilot comparison of t-NGS and conventional culture in severe suspected infections

**Figure 3:**
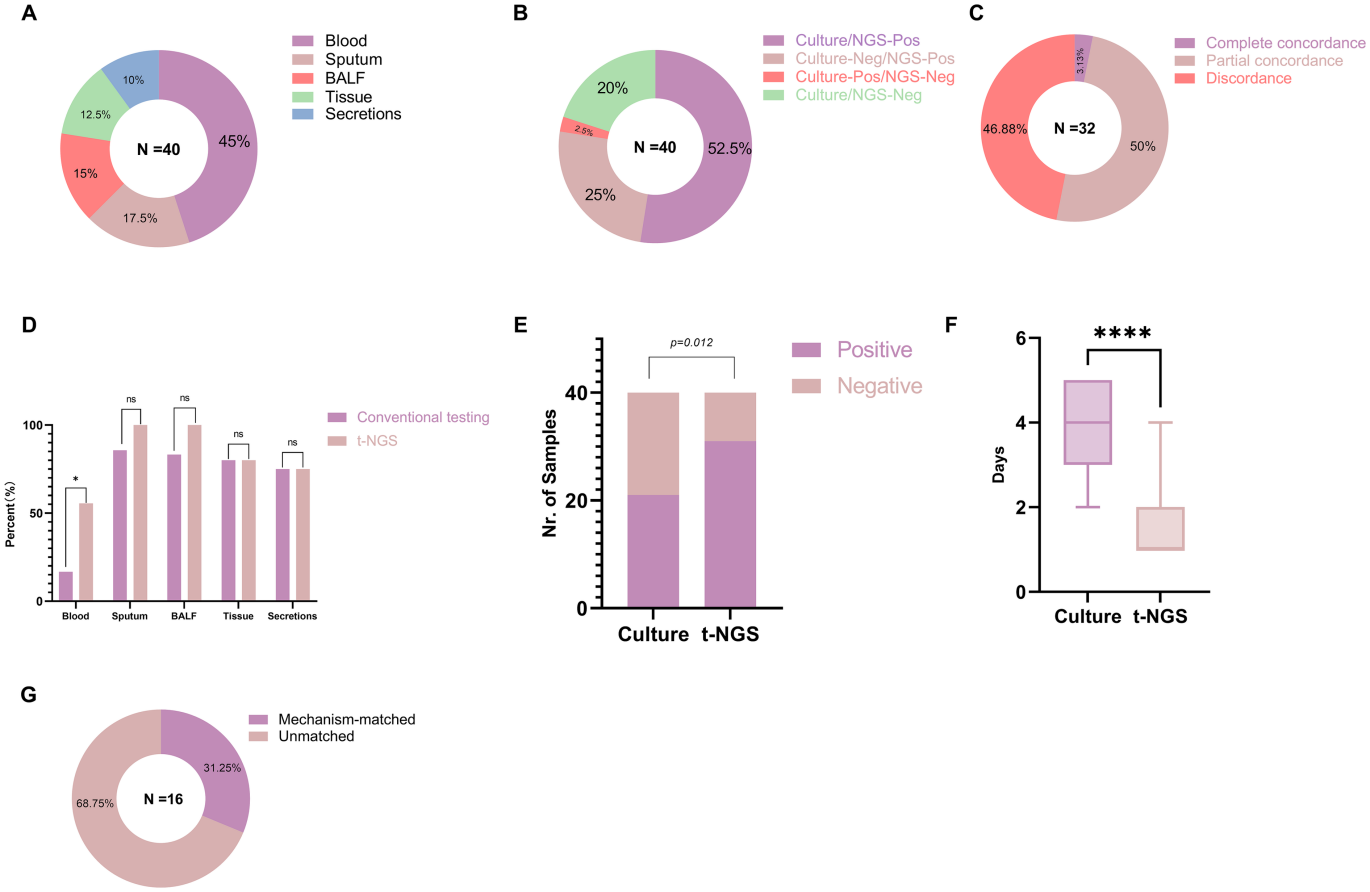
Comparison of conventional culture and t-NGS in pathogen detection. **(A)** Specimen type distribution. **(B)** Positive/negative consistency matrix between conventional culture and t-NGS. **(C)** Pathogen-level concordance among the 32 specimens positive by at least one method. **(D)** Detection rates by specimen type, with exact McNemar test comparisons. **(E)** Overall positivity rates. **(F)** Time to detection (median [IQR], Wilcoxon signed-rank test). **(G)** Mechanism-level genotype–phenotype concordance among 16 resistance-evaluable specimens. Paired detection analyses were performed on 40 specimens, whereas AMR genotype–phenotype concordance was assessed in 16 evaluable specimens.

A total of 40 paired clinical specimens were collected from 29 prospectively enrolled patients. Notably, these patients belonged to the highest severity stratum, with a mean TBSA of 73.93%. This group was consistent with the high-risk population defined in our retrospective baseline analysis. As illustrated in Figure 3A, blood samples constituted the predominant specimen type (18/40, 45%), followed by sputum (7/40, 17.5%), bronchoalveolar lavage fluid (6/40, 15%), tissue (5/40, 12.5%), and wound secretions (4/40, 10%). Comparative analysis of detection outcomes (Figure 3B) revealed that 52.5% (21/40) of samples were positive by both methods, while 20.0% (8/40) were negative by both. Discordant results included 25.0% (10/40) t-NGS-positive/culture-negative specimens and 2.5% (1/40) culture-positive/t-NGS-negative specimens. Accordingly, overall positivity was 77.5% (31/40) for t-NGS and 55.0% (22/40) for conventional culture (*p* = 0.012). Among the 32 specimens positive by at least one method, partial pathogen-level concordance was the most frequent outcome (16/32, 50.00%), followed by discordance (15/32, 46.88%) and complete concordance (1/32, 3.13%; Figure 3C). Stratification by specimen type (Figure 3D and Supplementary Table 7) highlighted performance disparities, particularly in blood specimens, where t-NGS showed 55.6% positivity (10/18) compared with 16.7% (3/18) for conventional culture (exact McNemar *p* = 0.039). These specimen-type findings are presented as a pilot subgroup analysis rather than definitive diagnostic validation. Overall positivity rates are summarized in Figure 3E. Furthermore, t-NGS significantly reduced the turnaround time (TAT) for pathogen identification, averaging 1.7 days compared to 3.9 days for conventional culture (*p* < 0.001; Figure 3F).

Microbiological analysis of the 32 positive specimens revealed a broad spectrum of microbial detections, including bacterial and fungal pathogens as well as viral DNA signals. Among bacteria, *Acinetobacter baumannii* was the most frequently detected organism, identified in 14 specimens, followed by *Klebsiella pneumoniae* in 11 specimens and *Stenotrophomonas maltophilia* in 9 specimens. *Pseudomonas aeruginosa* was detected in 5 specimens. Fungal detections included *Candida albicans* and *Candida parapsilosis*, each identified in 6 specimens, and *Candida tropicalis* in 4 specimens. For viral DNA detections, *human cytomegalovirus* was most frequent, detected in 14 specimens, followed by *human alphaherpesvirus 1* and *Epstein–Barr virus*, each detected in 6 specimens. The remaining organisms were mostly detected at low frequencies, generally in one to three specimens, reflecting a diverse microbial profile in t-NGS-positive samples (Supplementary Figure 4).

**Figure 4:**
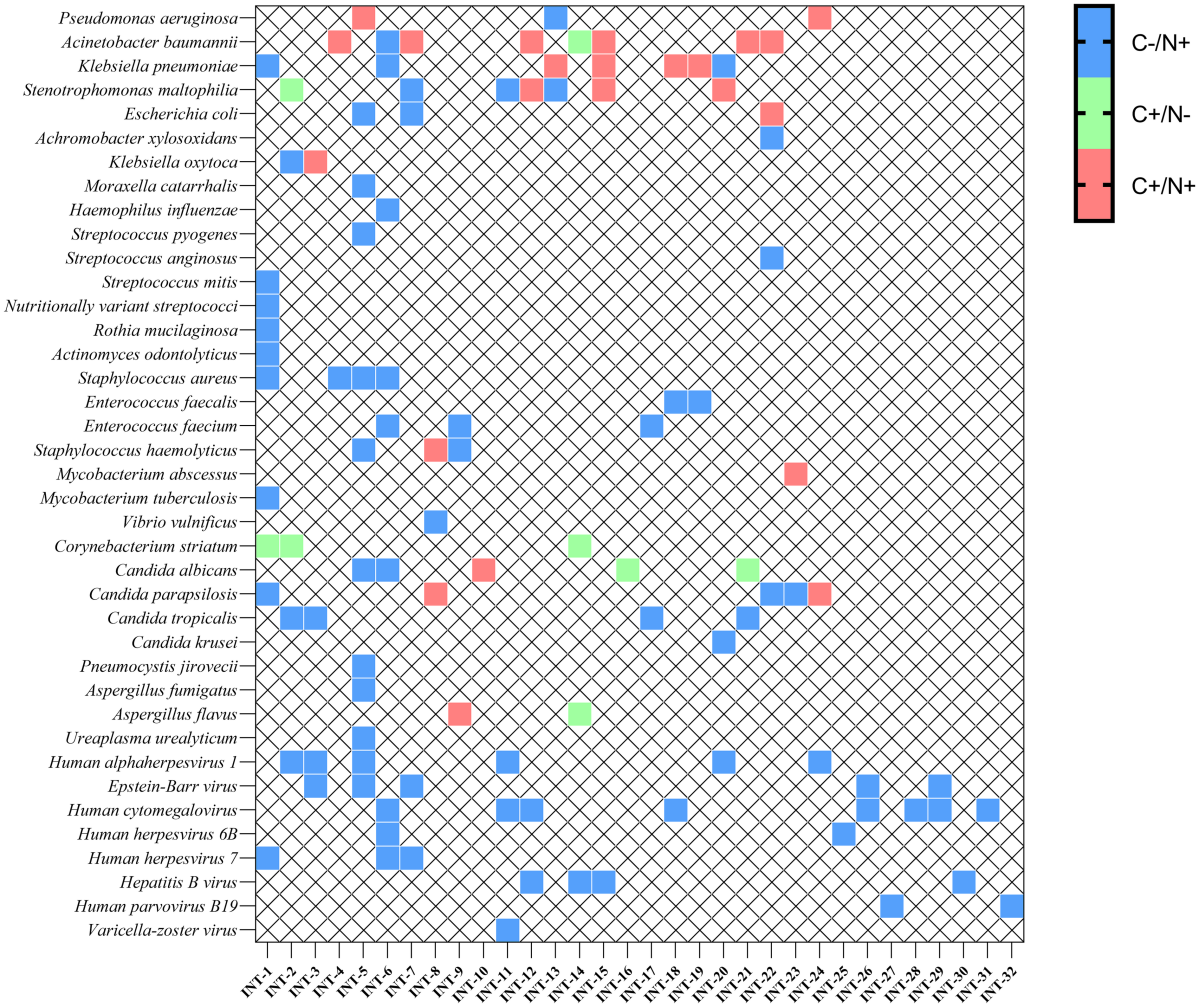
Pathogen identification profile across 32 positive samples using culture and t-NGS. Filled squares indicate detected pathogens. C: conventional culture; N: t-NGS; +: positive; −: negative; C+/N+: co-detection; C−/N+: t-NGS only; C+/N−: culture only.

Concordance analysis revealed that the “Culture-negative/t-NGS-positive” (C−/N+) pattern was the predominant observation (visually represented by blue blocks). Conversely, the “Culture-positive/t-NGS-negative” (C+/N−) pattern was rare, observed primarily in species such as Corynebacterium spp. Notably, high-prevalence pathogens, including *P. aeruginosa*, *A. baumannii*, and *C. albicans*, frequently demonstrated “C+/N+” co-detection (Figure 4).

To evaluate antimicrobial resistance, we screened the 32 pathogen-positive specimens, irrespective of their pathogen-detection concordance category. Phenotypic MDR patterns, mainly CRE, CRAB, CRPA, and methicillin-resistant staphylococci, were identified in 11 specimens, while AMR genes, including *mecA, OXA-23, OXA-48, KPC*, and *vanA*, were detected in 11 specimens. Sixteen specimens had a phenotypic MDR pattern, a detectable AMR gene, or both and were therefore considered resistance-evaluable. Mechanism-level concordance was defined as detection of an AMR gene capable of explaining the corresponding phenotypic resistance in the cultured organism. Five of the 16 evaluable specimens met this definition (31.25%), whereas the remaining 11 showed phenotype-only, gene-only, or non-matching phenotype–gene findings (68.75%; Figure 3G).

## 4 Discussion

Burn infection remains a major determinant of morbidity and mortality, particularly in patients with extensive burns. In this bicenter study from southern China, we demonstrated that burn severity, as quantified by TBSA, was closely associated with complication burden, pathogen spectrum, antimicrobial resistance patterns, and healthcare resource utilization.

Consistent with previous epidemiological studies (Zhang et al., 2021; Qu et al., 2023; Youssouf et al., 2024), male patients predominated across all burn severities, likely reflecting occupational exposure to high-risk environments involving flames, electricity, and chemicals (Brusselaers et al., 2010; McInnes et al., 2019). Age-specific clustering of burn etiology further highlighted young and middle-aged adults as the primary high-risk population for flame and electrical injuries, whereas scald injuries were more common at the extremes of age. These findings align with global burn registry data and reinforce the importance of targeted prevention strategies (Golshan et al., 2013; Albayrak et al., 2017; Pegg, 2005; Taylor et al., 2002; Mandelcorn et al., 2003).

Our descriptive analysis captured distinct seasonal variations in the monthly distribution of infected burn cases. Although this study cannot estimate population-level infection incidence or causal infection risk due to the absence of an uninfected control cohort, the descriptive clustering of extensive burn infections during specific months warrants clinical attention. In the humid, subtropical regions of Southern China, seasonal fluctuations in ambient temperature and humidity are known to interact with host physiology. These environmental factors can alter local tissue perfusion, modify oxygen delivery to the wound bed, and directly modulate bacterial proliferation (Cai et al., 2024).

In patients with mild burn infections (TBSA < 10%), burn sites were predominantly located on the lower and upper extremities, reflecting common accidental exposures encountered in daily activities. In contrast, patients with moderate to severe burns (TBSA ≥ 10%) exhibited a significantly higher proportion of multi-site involvement, suggesting that extensive thermal injury more readily overwhelms protective barriers. Perineal burns were consistently rare across all severity groups, a distribution pattern consistent with large-scale epidemiological surveys and likely attributable to anatomical protection and lower exposure risk (Panayi et al., 2024; Baba, 2022).

A graded association was observed between TBSA and the frequency of major complications among infected burn patients. Life-threatening conditions—including inhalation injury, shock, multiple organ dysfunction syndrome (MODS), and acute respiratory distress syndrome (ARDS)—were rare in patients with mild burns (TBSA < 10%) but escalated markedly in those with extensive burns (TBSA ≥ 50%). This finding aligns with data from the National Burn Repository and other multicenter cohorts (Silva et al., 2016; Lam and Minh, 2022; Miller et al., 2008; Bessey et al., 2014), likely reflecting the exacerbated systemic inflammatory response, endothelial dysfunction, and immune suppression associated with extensive thermal injury (Dries, 2009; Hemmila et al., 2008; Cartotto et al., 2024). Severe burn injury is characterized by profound systemic inflammation and immune dysregulation, which fundamentally alter host–pathogen interactions and contribute to the heterogeneity of post-burn infections. Emerging evidence from critically ill populations indicates that immune-related molecular signatures, such as S100A8/A9 and resistin, are closely associated with immune stratification and clinical outcomes (Chen, J. et al., 2025). This suggests that the host immune response plays a pivotal role in disease progression, extending beyond the impact of pathogen burden alone. Conversely, urinary tract infections showed no significant association with TBSA, implying that iatrogenic factors, rather than burn severity per se, are the primary drivers of this complication (Hemmila et al., 2008).

Hospitalization costs and length of stay increased progressively with burn severity, in line with prior cost analyses (Zeng et al., 2023; Abarca et al., 2023; Onah et al., 2021). The multivariate regression models in this study revealed that the number of surgical procedures was the strongest independent predictor associated with both higher hospitalization costs and prolonged LOS. This finding aligns with the prevailing consensus that surgical interventions in burn care—including escharotomy, serial debridement, and skin grafting—are inherently resource-intensive and closely associated with LOS (Hauc et al., 2024). Notably, although TBSA and inhalation injury markedly increased economic burdens, neither factor emerged as an independent predictor of prolonged LOS. This statistical dissociation indicates that the impact of initial anatomical injury severity on hospitalization duration is largely mediated by the subsequent requirement for repeated surgical procedures (Baraka et al., 2024). Accordingly, optimizing perioperative management and implementing standardized early definitive wound closure protocols may serve as key strategies to reduce healthcare expenditures and improve hospital bed turnover efficiency simultaneously. Another noteworthy finding was that positive microbial detection on initial admission was independently associated with reduced healthcare costs and shorter LOS. Although seemingly counterintuitive, this negative correlation likely reflects the substantial clinical benefits of early microbial screening. Prompt pathogen identification at admission enables clinicians to initiate targeted antimicrobial therapy and implement strict isolation protocols without delay. This proactive strategy prevents the development of insidious, late-onset healthcare-associated infections (HAIs) and uncontrolled septic shock, complications that typically trigger substantial healthcare cost escalation and persistent discharge delays (Ryu et al., 2023).

Microbiological analysis revealed a marked shift in infection patterns with increasing burn severity. While single-pathogen infections predominated in mild burns, polymicrobial infections became more frequent in severe burns, likely driven by immune dysfunction, prolonged hospitalization, and increased invasive procedures (Gong et al., 2021). Concomitantly, pathogen composition shifted from Gram-positive dominance toward Gram-negative bacteria and fungi as TBSA increased. In particular, *Acinetobacter baumannii* and *Klebsiella pneumoniae* were significantly enriched in patients with TBSA ≥ 50%, consistent with their role as major nosocomial pathogens in burn ICUs (Fuentes-González et al., 2024; Maitz et al., 2023; Maslova et al., 2021) The rising proportion of fungal infections further reflects immune suppression and broad-spectrum antibiotic exposure in extensively burned patients (Nitsani et al., 2023).

Furthermore, the chronological timing of specimen collection represents a critical variable in post-burn microbial ecology. In our cohort, the distinct microbial transition from Gram-positive dominance (63.96%) in mild burns to Gram-negative (56.62%) and fungal (20.62%) enrichment in severe burns is inherently linked to the clinical timeline of trauma care. Patients with extensive injuries (≥50% TBSA) required a markedly protracted median hospitalization of 58 days, compared to only 19 days for the mild cohort. This extended clinical window inevitably subjected severe burn patients to prolonged nosocomial exposure, multiple invasive interventions, and sequential broad-spectrum antibiotic therapies (Gong et al., 2021; Rout et al., 2025). Consequently, cultures from the high-TBSA group naturally encapsulated late-stage, secondary healthcare-associated infections, which are conventionally dominated by multi-drug resistant Gram-negative bacilli and opportunistic fungi. Although the retrospective and event-driven nature of our clinical registry limits the precise chronological pairing of every isolate to exact post-burn hours, this temporal progression underscores that burn severity and hospitalization duration synergistically shape the shifting infectious landscape.

Antimicrobial resistance patterns exhibited a strong positive association with burn severity. Gram-negative pathogens in the TBSA ≥ 50% group demonstrated high resistance rates to multiple antibiotic classes, particularly carbapenems, mirroring national and international trends in burn centers (Robben et al., 2021; Ruegsegger et al., 2022; Elsheikh and Makram, 2024). Among Gram-positive bacteria, resistance rates of Staphylococcus species increased with TBSA, although susceptibility to glycopeptides and linezolid remained preserved (Roy et al., 2024; Yang et al., 2024; Mozafari et al., 2024). Notably, *Candida tropicalis* exhibited substantially higher azole resistance than *Candida albicans*, highlighting the growing challenge of antifungal resistance in severe burn infections (Xiong et al., 2025; Obenhuber et al., 2024).

Beyond individual patient factors, the profound shift toward MDR Gram-negative bacteria in severe burns is inextricably linked to the intense ecological resistance pressures within the burn ICU environment. Extensively burned patients serve as prolonged reservoirs for multidrug-resistant organisms. The routine empirical administration of broad-spectrum antimicrobials, combined with the presence of multiple invasive devices, exerts tremendous selective pressure (Nasser et al., 2025). Furthermore, the specialized but highly trafficked nature of burn ICUs facilitates environmental contamination and cross-transmission of highly resilient pathogens, such as CRAB and CRE, which readily form biofilms on both necrotic eschar and abiotic hospital surfaces (Elsheikh and Makram, 2024). Therefore, the resistance profiles observed in our severe cohort are not merely biological consequences of thermal injury, but rather a reflection of the complex ICU microecology.

Overall, this bicenter study from southern China highlights a strong association between burn severity and pathogen resistance patterns, shaped not only by patient-related factors but also by hospital ecology and antibiotic utilization practices (Maharjan et al., 2026). These findings underscore the need for severity-stratified antimicrobial strategies and strengthened infection control measures in patients with extensive burns to improve outcomes and reduce healthcare burden (Macesic et al., 2025). Future studies integrating advanced molecular diagnostics and pharmacokinetic approaches are warranted to better address the growing challenge of antimicrobial resistance in burn care (Alharthi et al., 2025).

The prospective comparison between conventional culture and t-NGS suggested that, in a selected pilot cohort of severe suspected infections, t-NGS provided a higher detection yield and shorter turnaround time than culture. This analysis should be interpreted as an adjunct diagnostic comparison rather than definitive validation across all burn infections, and molecular findings must be interpreted alongside clinical phenotypes. These findings corroborate recent reports supporting the utility of sequencing-based diagnostics in complex and culture-negative infections (Liu et al., 2024; Zhou et al., 2023; Chen, W. et al., 2025; Yang et al., 2022; Rimoldi et al., 2023). Moreover, partial concordance between resistance genes detected by t-NGS and phenotypic MDR patterns suggests a complementary role for t-NGS in guiding early antimicrobial decision-making, although challenges related to result interpretation remain (Tian et al., 2023).

However, a critical aspect of implementing t-NGS in burn care is interpreting discordant results between molecular and conventional methods. In our cohort, discordance was predominantly characterized by a “culture-negative/t-NGS-positive” (C−/N+) pattern. In the absence of a true, sterile ‘gold standard’ for complex burn wounds, this higher positivity rate does not inherently represent superior diagnostic accuracy. This discrepancy is largely driven by the biological realities of severe burn management: patients routinely receive massive empirical antibiotic therapy prior to sampling, which can rapidly render invasive pathogens non-viable or induce a viable but non-culturable (VBNC) state (Chen, Q. et al., 2025). While conventional culture relies on living organisms, t-NGS is highly susceptible to amplifying residual nucleic acids from these dead pathogens, as well as bystander nucleic acids from localized microbial colonization or trace environmental contaminants. Although our protocol implemented strict clinical adjudication, the potential for false-positives remains, and the real-time impact of t-NGS results on altering clinical antibiotic management was not systematically tracked. Conversely, the “culture-positive/t-NGS-negative” (C+/N-) pattern was rare but notable, particularly among specific Gram-positive species such as *Corynebacterium* spp. This specific discordance highlights inherent technical bottlenecks: organisms with robust peptidoglycan cell walls may resist standard lysis protocols, leading to suboptimal nucleic acid extraction (Scanlon et al., 2025). Furthermore, pathogens present at extremely low abundances in complex polymicrobial burn wounds may fall below the stringent bioinformatic read-count thresholds designed to filter out background noise, resulting in negative molecular reports (Halford et al., 2024).

Second, the relatively low concordance (31.25%) observed between the detected resistance genes and phenotypic MDR status highlights the technical gap between genotype and phenotype. The presence of an AMR gene does not guarantee its functional expression under antimicrobial pressure (Centner et al., 2026). Conversely, phenotypic resistance in critical burn pathogens—such as *Pseudomonas aeruginosa* or *Acinetobacter baumannii*—is often mediated by alternative mechanisms like efflux pump upregulation or membrane porin alterations, which are missed by targeted gene sequencing panels (Rizvanoglu et al., 2026). Additionally, within polymicrobial specimens, t-NGS cannot definitively link a specific resistance gene to its host organism. Similarly, the identification of viral DNA, such as Human alphaherpesvirus 1, likely reflects transient viral reactivation driven by post-burn immunosuppression rather than active viral infection (Roy et al., 2024). Therefore, t-NGS should be utilized as a complementary tool alongside traditional culture to potentially inform early targeted antimicrobial therapy. Future prospective interventional trials with predefined stewardship metrics (e.g., days of therapy, mortality) are required to confirm its definitive clinical impact.

Collectively, our findings emphasize that burn severity, compounded by subsequent healthcare exposure intensity, is closely associated with infectious complications, pathogen distribution, and resistance burden. The integration of advanced molecular diagnostics such as t-NGS with conventional microbiological methods may facilitate earlier pathogen identification in selected patients with extensive burns and suspected systemic infections, but larger prospective studies are needed before broad diagnostic superiority can be concluded.

## 5 Conclusion

Several limitations of this study should be acknowledged. Structurally, the retrospective cohort design constraints causal inference and leaves the baseline incidence of infections unestimated due to the absence of uninfected controls. Concurrently, potential heterogeneity in case ascertainment may exist as diagnoses relied on existing clinical records rather than protocol-driven criteria across the two centers. Regarding confounding variables, systematic adjustments for healthcare-associated confounders—including prolonged length of stay, multiple surgical interventions, invasive device utilization, and prior broad-spectrum antibiotic or prophylactic antifungal exposures—were unfeasible, which may cloud the observed resistance trajectories—particularly for azole-resistant non-albicans Candida species. Furthermore, the prospective t-NGS validation was limited by a small sample size, rendering it underpowered for robust clinical outcome verification. Technologically, neither method reliably delineates active infection from colonization at non-sterile burn sites. Lastly, given that data were sourced exclusively from two tertiary centers in southern China, the subtropical epidemiological patterns—such as elevated fungal prevalence—may not fully generalize to disparate geographic climates, while the cross-sectional nature of resistance tracking precludes mapping dynamic long-term stewardship trends. In summary, this bicenter study demonstrates a strong, severity-dependent association between TBSA and the epidemiology of burn infections—specifically the increased prevalence of polymicrobial, Gram-negative, and fungal infections in severe cases—and characterizes MDR patterns to inform risk-adapted management strategies. Furthermore, the pilot integration of t-NGS suggested enhanced detection yield and efficiency in selected severe suspected infections, particularly in culture-negative scenarios. From a clinical perspective, these advancements advocate for precise, early interventions and optimized antibiotic stewardship. Ultimately, while these approaches may support earlier risk-adapted management and antibiotic stewardship, further large-scale prospective interventional trials are warranted to verify whether routine t-NGS integration improves patient outcomes, bed turnover, or economic burden in severe burn care.

## Data Availability

The raw data supporting the conclusions of this article will be made available by the authors, without undue reservation.

## Footnotes

1
https://www.ncbi.nlm.nih.gov/genome
