# Supplementary figures and images for: Epidemiology, pathogen spectrum, and antimicrobial resistance of infections in burn patients stratified by total body surface area: a bicenter study with evaluation of targeted next-generation sequencing
Source: Front Microbiol. 2026 Jul 15;17:1866146. doi: 10.3389/fmicb.2026.1866146 (PMC13415348; doi:10.3389/fmicb.2026.1866146)

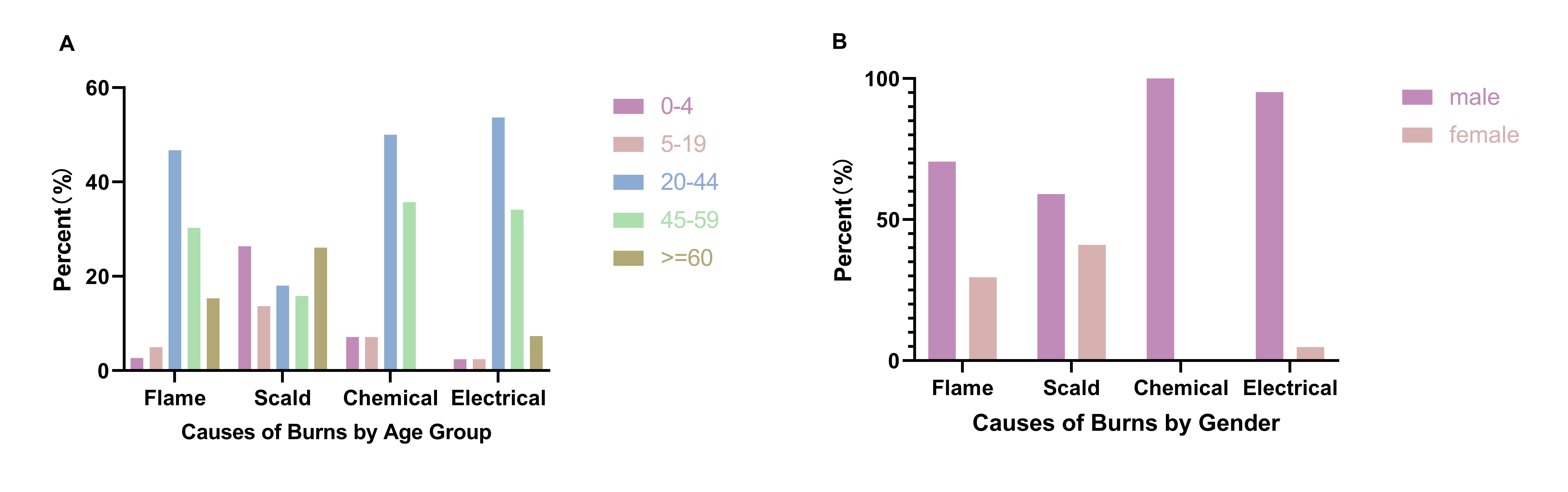

Supplement: Supplementary file 1 [file Image_1.TIF]

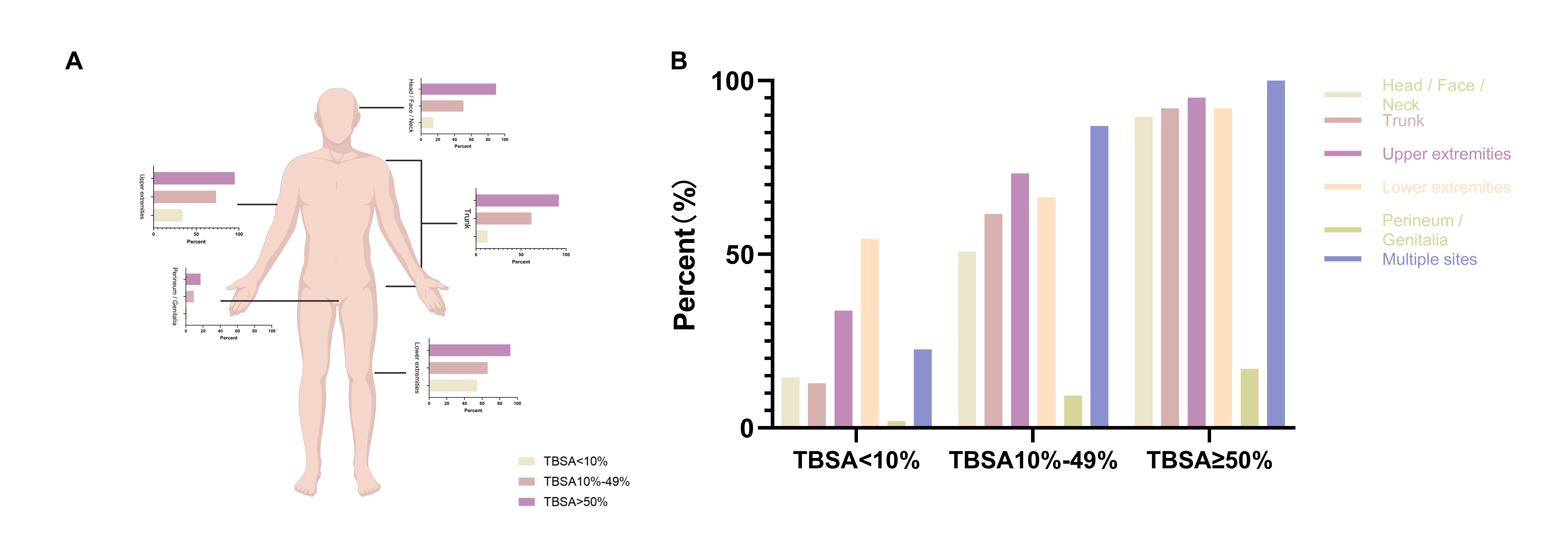

Supplement: Supplementary file 2 [file Image_2.TIF]

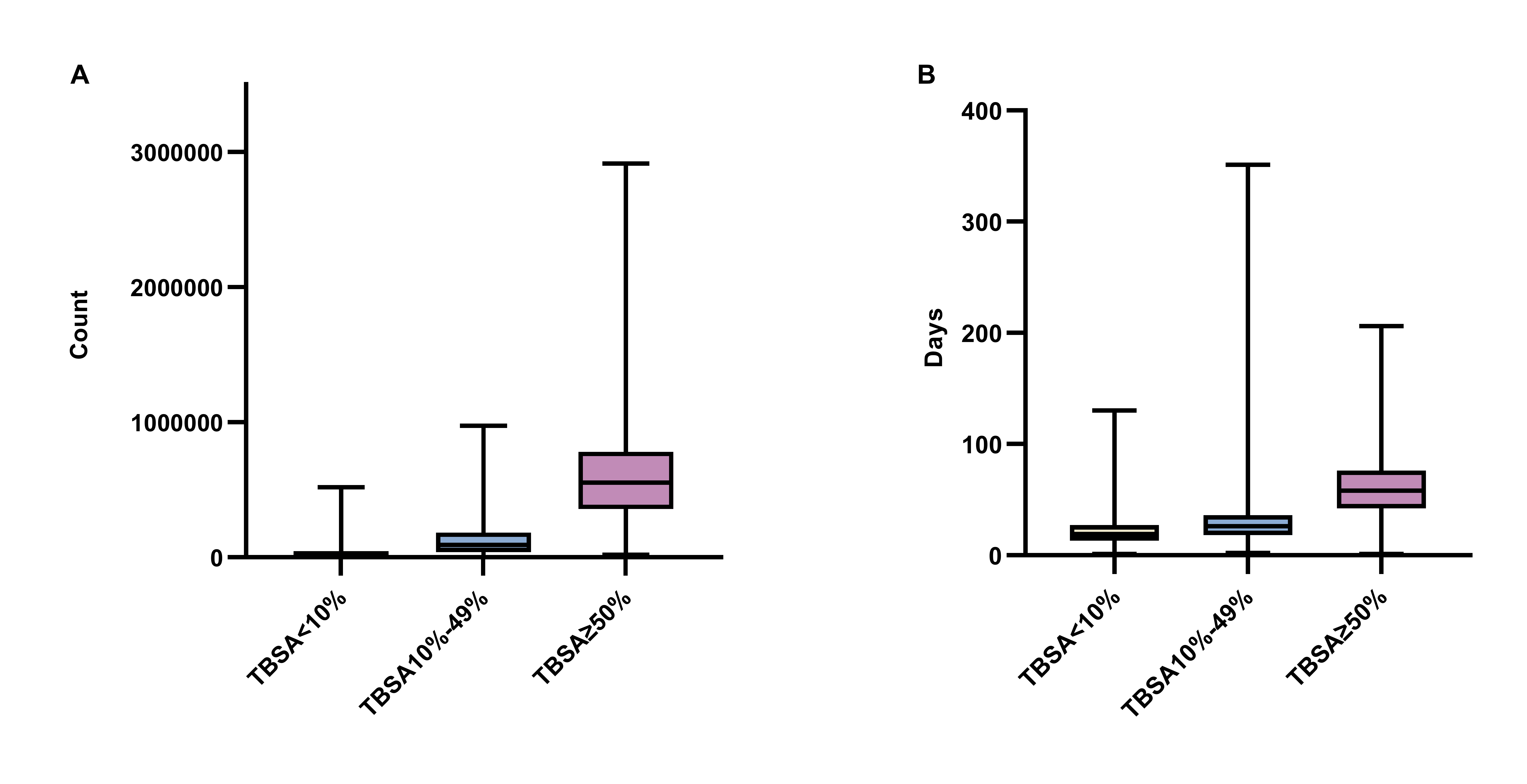

Supplement: Supplementary file 3 [file Image_3.TIF]

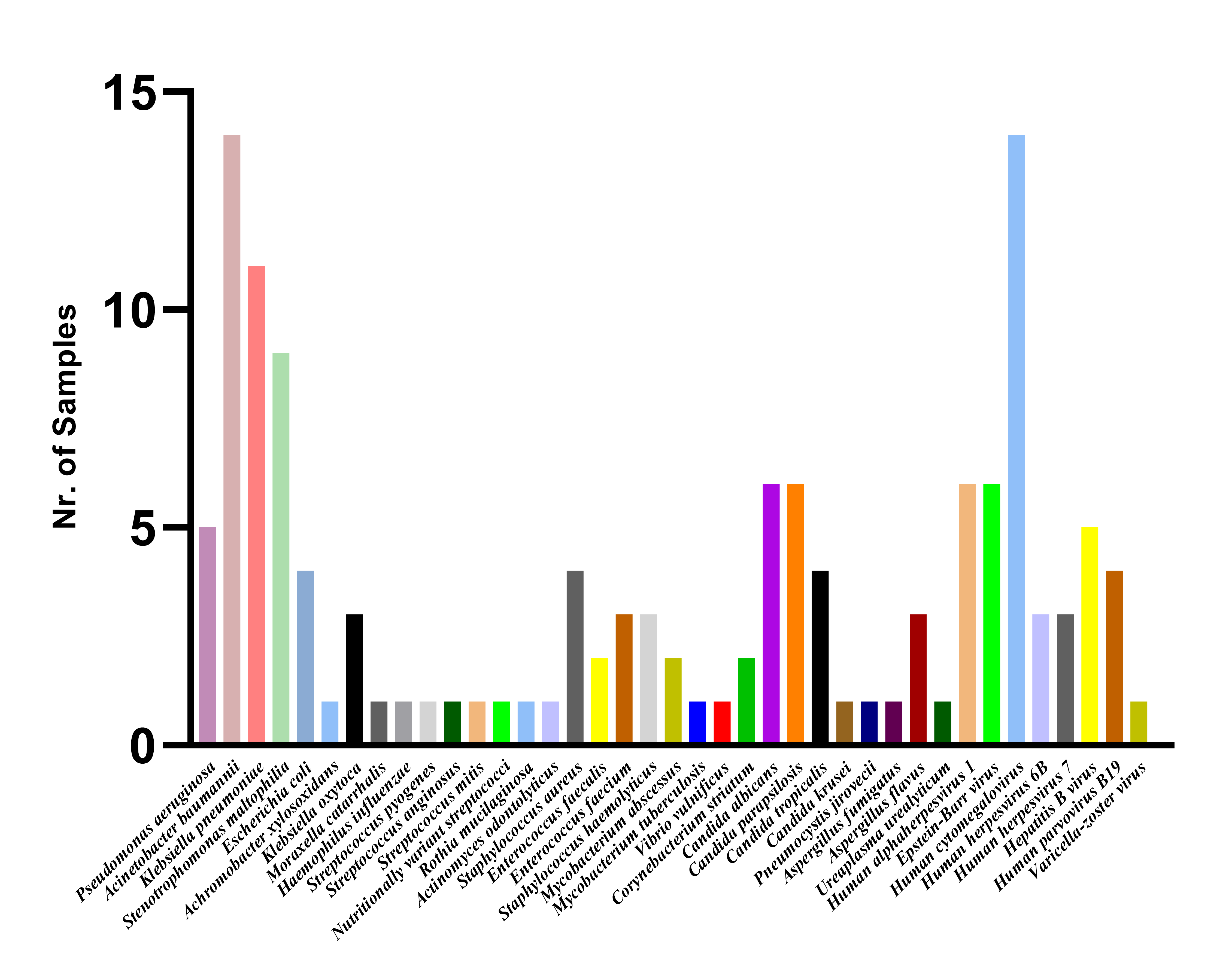

Supplement: Supplementary file 4 [file Image_4.TIF]
