# Supplementary material for: Epidemiology, pathogen spectrum, and antimicrobial resistance of infections in burn patients stratified by total body surface area: a bicenter study with evaluation of targeted next-generation sequencing
Source: Front Microbiol. 2026 Jul 15;17:1866146. doi: 10.3389/fmicb.2026.1866146 (PMC13415348; doi:10.3389/fmicb.2026.1866146)
Supplement: Supplementary file 5 [file Table_1.DOCX]

| **Supplementary Table 1** Frequency of complications among infected burn patients | | | | | | | |
| --- | --- | --- | --- | --- | --- | --- | --- |
|  | TBSA<10%  n=358 | | TBSA10%-49%  n=292 | | TBSA≥50%  n=164 | | *P* value |
|  | Number | Percent(%) | Number | Percent(%) | Number | Percent(%) |  |
| Inhalation Injury | 12 | 3.35 | 71 | 24.32 | 126 | 76.83 | <0.001 |
| shock | 3 | 0.84 | 71 | 24.32 | 116 | 70.73 | <0.001 |
| Compartment Syndrome | 1 | 0.28 | 26 | 8.90 | 75 | 45.73 | <0.001 |
| Wound Infection | 105 | 29.33 | 55 | 18.84 | 53 | 32.32 | 0.001 |
| MODS | 0 | 0.00 | 11 | 3.77 | 48 | 29.27 | <0.001 |
| Pneumonia | 25 | 6.98 | 21 | 7.19 | 29 | 17.68 | <0.001 |
| ARDS | 0 | 0.00 | 3 | 1.03 | 28 | 17.07 | <0.001 |
| Bloodstream Infection | 0 | 0.00 | 8 | 2.74 | 26 | 15.85 | <0.001 |
| Electrolyte and Protein Disorder | 8 | 2.23 | 13 | 4.45 | 25 | 15.24 | <0.001 |
| Acute Kidney Injury | 1 | 0.28 | 5 | 1.71 | 24 | 14.63 | <0.001 |
| Systemic Inflammatory Response Syndrome | 0 | 0.00 | 4 | 1.37 | 8 | 4.88 | <0.001 |
| PTSD | 3 | 0.84 | 3 | 1.03 | 7 | 4.27 | 0.009 |
| Urinary Tract Infection | 8 | 2.23 | 4 | 1.37 | 5 | 3.05 | 0.469 |
| *MODS:Multiple Organ Dysfunction Syndrome. ARDS: Acute Respiratory Distress Syndrome. PTSD: Post-Traumatic Stress Disorder.* | | | | | | | |
|  |  |  |  |  |  |  |  |

| Suppl. Table 2. Clinical characteristics of infected burn patients stratified by total body surface area | | | | | | | |
| --- | --- | --- | --- | --- | --- | --- | --- |
|  | TBSA<10% n=358 | | TBSA10%-49% n=292 | | TBSA≥50% n=164 | | *P* value |
|  | Number | Percent(%) | Number | Percent(%) | Number | Percent(%) |  |
| Smoking status |  |  |  |  |  |  | 0.044 |
| yes | 13 | 3.63 | 17 | 5.82 | 7 | 4.27 |  |
| No | 320 | 89.39 | 244 | 83.56 | 151 | 92.07 |  |
| Unknown | 25 | 6.98 | 31 | 10.62 | 6 | 3.66 |  |
| Inhalation injury |  |  |  |  |  |  | <0.001 |
| Yes | 12 | 3.35 | 71 | 24.32 | 126 | 76.83 |  |
| No | 323 | 90.22 | 221 | 75.68 | 38 | 23.17 |  |
| Unknown | 23 | 6.42 | 0 | 0.00 | 0 | 0.00 |  |
| Tracheal intubation |  |  |  |  |  |  | <0.001 |
| Yes | 51 | 14.25 | 75 | 25.68 | 93 | 56.71 |  |
| No | 288 | 80.45 | 213 | 72.95 | 71 | 43.29 |  |
| Unknown | 19 | 5.31 | 4 | 1.37 |  | 0.00 |  |
| Number of surgeries |  |  |  |  |  |  | <0.001 |
| 0 | 146 | 40.78 | 71 | 24.32 | 9 | 5.49 |  |
| 1 time | 119 | 33.24 | 74 | 25.34 | 3 | 1.83 |  |
| 2-4 times | 86 | 24.02 | 129 | 44.18 | 64 | 39.02 |  |
| ≥5 times | 7 | 1.96 | 18 | 6.16 | 88 | 53.66 |  |
| Treatment outcome |  |  |  |  |  |  | <0.001 |
| Cured | 333 | 93.02 | 271 | 92.81 | 129 | 78.66 |  |
| Partially cured | 10 | 2.79 | 12 | 4.11 | 18 | 10.98 |  |
| Not cured | 2 | 0.56 | 8 | 2.74 | 6 | 3.66 |  |
| Death | 0 | 0.00 | 1 | 0.34 | 11 | 6.71 |  |
| Unknown | 13 | 3.63 |  | 0.00 |  | 0.00 |  |
| Underlying diseases |  |  |  |  |  |  |  |
| Cardiovascular diseases | 62 | 17.32 | 23.00 | 7.88 | 8.00 | 4.88 | <0.001 |
| Diabetes | 56 | 15.64 | 10.00 | 3.42 | 4.00 | 2.44 | <0.001 |
| Cerebrovascular diseases (stroke、cerebral apoplexy) | 8 | 2.23 | 2.00 | 0.68 | 0.00 | 0.00 | 0.057 |
| Neurological diseases (epilepsy、Alzheimer's disease) | 9 | 2.51 | 3.00 | 1.03 | 0.00 | 0.00 | 0.063 |
| Kidney disease | 6 | 1.68 | 4.00 | 1.37 | 2.00 | 1.22 | 0.907 |
| Tumor / Cancer | 3 | 0.84 | 4.00 | 1.37 | 0.00 | 0.00 | 0.314 |
| Mental illness / Mental disorder | 0 | 0.00 | 0.00 | 0.00 | 1.00 | 0.61 | 0.137 |

| **Supplementary Table 3** Distribution of bacterial and fungal isolates among burn patients stratified by TBSA | | | | | | | |
| --- | --- | --- | --- | --- | --- | --- | --- |
| Bacterial species | **TBSA<10%**  **n=591** | | **TBSA10-49%**  **n=415** | | **TBSA>=50%**  **n=650** | | *P*-value |
|  | Count | Percentage（%） | Count | Percentage（%） | Count | Percentage（%） |  |
| Gram-positive bacteria | **378** | 63.96 | **215** | 51.81 | **148** | 22.77 | **<0.001** |
| *Staphylococcus aureus* | 131 | 22.17 | 38 | 9.16 | 11 | 1.69 | <0.001 |
| *Coagulase-negative staphylococci* | 28 | 4.74 | 26 | 6.27 | 25 | 3.85 | 0.196 |
| *Staphylococcus epidermidis* | 80 | 13.54 | 34 | 8.19 | 30 | 4.62 | <0.001 |
| *Enterococcus faecium* | 38 | 6.43 | 39 | 9.40 | 13 | 2.00 | <0.001 |
| *Hemolytic staphylococci* | 13 | 2.20 | 17 | 4.10 | 15 | 2.31 | 0.136 |
| *Corynebacterium spp.* | 17 | 2.88 | 11 | 2.65 | 20 | 3.08 | 0.921 |
| *Enterococcus faecalis* | 3 | 0.51 | 9 | 2.17 | 11 | 1.69 | 0.060 |
| *Bacillus cereus* | 7 | 1.18 | 6 | 1.45 | 6 | 0.92 | 0.733 |
| *Streptococcus pyogenes* | 4 | 0.68 | 0 | 0.00 | 0 | 0.00 | 0.027 |
| Others | 57 | 9.64 | 35 | 8.43 | 17 | 2.62 | - |
| Gram-negative bacteria | **160** | 27.07 | **142** | 34.22 | **368** | 56.62 | <0.001 |
| *Pseudomonas aeruginosa* | 41 | 6.94 | 34 | 8.19 | 60 | 9.23 | 0.337 |
| *Acinetobacter baumannii* | 25 | 4.23 | 29 | 6.99 | 92 | 14.15 | <0.001 |
| *Klebsiella pneumoniae* | 14 | 2.37 | 19 | 4.58 | 123 | 18.92 | <0.001 |
| *Escherichia coli* | 21 | 3.55 | 25 | 6.02 | 19 | 2.92 | 0.033 |
| *Enterobacter cloacae* | 11 | 1.86 | 10 | 2.41 | 15 | 2.31 | 0.805 |
| *Stenotrophomonas maltophilia* | 7 | 1.18 | 3 | 0.72 | 26 | 4.00 | <0.001 |
| Other | 41 | 6.94 | 22 | 5.30 | 33 | 5.08 | - |
| Fungi | **53** | 8.97 | **58** | 13.98 | **134** | 20.62 | <0.001 |
| *Candida parapsilosis* | 23 | 3.89 | 18 | 4.34 | 16 | 2.46 | 0.198 |
| *Candida albicans* | 15 | 2.54 | 11 | 2.65 | 34 | 5.23 | 0.019 |
| *Candida tropicalis* | 7 | 1.18 | 10 | 2.41 | 37 | 5.69 | <0.001 |
| *Candida glabrata* | 4 | 0.68 | 5 | 1.20 | 9 | 1.38 | 0.469 |
| *Aspergillus flavus* | 0 | 0.00 | 3 | 0.72 | 12 | 1.85 | 0.003 |
| *Aspergillus fumigatus* | 0 | 0.00 | 2 | 0.48 | 7 | 1.08 | 0.035 |
| Other | 4 | 0.68 | 9 | 2.17 | 19 | 2.92 | - |

**Supplementary Table 4** Antimicrobial resistance rates of major Gram-negative bacteria stratified by TBSA

|  | ***Acinetobacter baumannii*** | | | ***Pseudomonas aeruginosa*** | | | ***Klebsiella pneumoniae*** | | | ***Escherichia coli*** | | |
| --- | --- | --- | --- | --- | --- | --- | --- | --- | --- | --- | --- | --- |
| Antimicrobial resistance | TBSA<10%  n=25 | TBSA10-49%  n=29 | TBSA>=50%  n=92 | TBSA<10%  n=41 | TBSA10-49%  n=34 | TBSA>=50%  n=60 | TBSA<10%  n=14 | TBSA10-49%  n=19 | TBSA>=50%  n=123 | TBSA<10%  n=21 | TBSA10-49%  n=25 | TBSA>=50%  n=19 |
| Ceftriaxone | - | - | - | - | - | - | 5(35.71%) | 12(63.16%) | 109(88.62%) | 16(76.19%) | 20(80.00%) | 15(78.95%) |
| Ceftazidime | 10(40.00%) | 19(65.52%) | 84(91.30%) | 2(4.88%) | 6(17.65%) | 20(33.33%) | 5(35.71%) | 10(52.63%) | 94(76.42%) | 7(33.33%) | 13(52.00%) | 4(21.05%) |
| Cefepime | 9(36.00%) | 18(62.07%) | 80(86.96%) | 2(4.88%) | 2(5.88%) | 15(25.00%) | 5(35.71%) | 10(52.63%) | 96(78.05%) | 7(33.33%) | 13(52.00%) | 6(31.58%) |
| Cefotaxime | - | - | - | - | - | - | 5(35.71%) | 13(68.42%) | 109(88.62%) | 16(76.19%) | 20(80.00%) | 15(78.95%) |
| Cefoperazone–sulbactam | 9(36.00%) | 19(65.52%) | 81(88.04%) | 2(4.88%) | 8(23.53%) | 18(30.00%) | 4(28.57%) | 8(42.11%) | 87(70.73%) | 0(0.00%) | 5(20.00%) | 1(5.26%) |
| Piperacillin–tazobactam | 9(36.00%) | 19(65.52%) | 85(92.39%) | 3(7.32%) | 7(20.59%) | 13(21.67%) | 2(14.29%) | 6(31.58%) | 91(73.98%) | 0(0.00%) | 5(20.00%) | 1(5.26%) |
| Meropenem | 8(32.00%) | 19(65.52%) | 84(91.30%) | 1(2.44%) | 6(17.65%) | 20(33.33%) | 1(7.14%) | 6(31.58%) | 85(69.11%) | 0(0.00%) | 3(12.00%) | 1(5.26%) |
| Imipenem | 9(36.00%) | 19(65.52%) | 84(91.30%) | 4(9.76%) | 7(20.59%) | 21(35.00%) | 1(7.14%) | 6(31.58%) | 85(69.11%) | 0(0.00%) | 3(12.00%) | 1(5.26%) |
| Amikacin | 8(32.00%) | 17(58.62%) | 86(93.48%) | 1(2.44%) | 3(8.82%) | 5(8.33%) | 2(14.29%) | 7(36.84%) | 82(66.67%) | 0(0.00%) | 1(4.00%) | 2(10.53%) |
| Gentamicin | 8(32.00%) | 19(65.52%) | 83(90.22%) | 3(7.32%) | 6(17.65%) | 13(21.67%) | 3(21.43%) | 9(47.37%) | 100(81.30%) | 7(33.33%) | 7(28.00%) | 15(78.95%) |
| Ciprofloxacin | 9(36.00%) | 19(65.52%) | 83(90.22%) | 7(17.07%) | 6(17.65%) | 14(23.33%) | 10(71.43%) | 12(63.16%) | 120(97.56%) | 13(61.90%) | 18(72.00%) | 14(73.68%) |
| Levofloxacin | 10(40.00%) | 19(65.52%) | 85(92.39%) | 8(19.51%) | 9(26.47%) | 19(31.67%) | 7(50.00%) | 11(57.89%) | 107(86.99%) | 12(57.14%) | 17(68.00%) | 12(63.16%) |
| Colistin | 0(0.00%) | 1(3.45%) | 0(0.00%) | 0(0.00%) | 1(2.94%) | 0(0.00%) | - | - | - | - | - | - |
| Trimethoprim–sulfamethoxazole | 14(56.00%) | 20(68.97%) | 82(89.13%) | - | - | - | 10(71.43%) | 15(78.95%) | 115(93.50%) | 13(61.90%) | 19(76.00%) | 18(94.74%) |
| Tetracycline | 11(44.00%) | 19(65.52%) | 80(86.96%) | - | - | - | 11(78.57%) | 13(68.42%) | 111(90.24%) | 14(66.67%) | 20(80.00%) | 13(68.42%) |
| Minocycline | 6(24.00%) | 5(17.24%) | 23(25.00%) | - | - | - | 4(28.57%) | 7(36.84%) | 70(56.91%) | 1(4.76%) | 7(28.00%) | 1(5.26%) |
| Ampicillin–sulbactam | 9(36.00%) | 19(65.52%) | 83(90.22%) | - | - | - | 9(64.29%) | 9(47.37%) | 103(83.74%) | 8(38.10%) | 13(52.00%) | 10(52.63%) |

**Supplementary Table 5** Antimicrobial resistance rates of major Gram-positive bacteria stratified by TBSA

|  | *Staphylococcus aureus* | | | *Staphylococcus epidermidis* | | | *Enterococcus faecium* | | |
| --- | --- | --- | --- | --- | --- | --- | --- | --- | --- |
| Antimicrobial resistance | TBSA<10%  n=131 | TBSA10-49%  n=38 | TBSA>=50%  n=11 | TBSA<10%  n=80 | TBSA10-49%  n=34 | TBSA>=50%  n=30 | TBSA<10%  n=38 | TBSA10-49%  n=39 | TBSA>=50%  n=13 |
| Penicillin | 121(92.37%) | 36(94.74%) | 11(100.00%) | 70(87.50%) | 34(100.00%) | 30(100.00%) | 1(2.63%) | 0(0.00%) | 0(0.00%) |
| Oxacillin | 28(21.37%) | 16(42.11%) | 8(72.73%) | 44(55.00%) | 34(100.00%) | 30(100.00%) | - | - | - |
| Vancomycin | 0(0.00%) | 0(0.00%) | 0(0.00%) | 0(0.00%) | 0(0.00%) | 0(0.00%) | 0(0.00%) | 0(0.00%) | 0(0.00%) |
| Linezolid | 0(0.00%) | 0(0.00%) | 0(0.00%) | 0(0.00%) | 0(0.00%) | 0(0.00%) | 1(2.63%) | 0(0.00%) | 0(0.00%) |
| Teicoplanin | 0(0.00%) | 0(0.00%) | 0(0.00%) | 0(0.00%) | 0(0.00%) | 0(0.00%) | 0(0.00%) | 0(0.00%) | 0(0.00%) |
| Clindamycin | 48(36.64%) | 18(47.37%) | 8(72.73%) | 26(32.50%) | 10(29.41%) | 7(23.33%) | - | - | - |
| Erythromycin | 52(39.69%) | 18(47.37%) | 8(72.73%) | 46(57.50%) | 15(44.12%) | 7(23.33%) | 21(55.26%) | 18(46.15%) | 8(61.54%) |
| Trimethoprim–sulfamethoxazole | 5(3.82%) | 4(10.53%) | 3(27.27%) | 23(28.75%) | 15(44.12%) | 21(70.00%) | - | - | - |
| Tetracycline | 25(19.08%) | 13(34.21%) | 6(54.55%) | 4(5.00%) | 15(44.12%) | 4(13.33%) | 21(55.26%) | 30(76.92%) | 10(76.92%) |
| Gentamicin | 9(6.87%) | 6(15.79%) | 7(63.64%) | 2(2.50%) | 5(14.71%) | 16(53.33%) | - | - | - |
| Ciprofloxacin | 14(10.69%) | 7(18.42%) | 10(90.91%) | 22(27.50%) | 15(44.12%) | 23(76.67%) | 5(13.16%) | 12(30.77%) | 3(23.08%) |
| Levofloxacin | 10(7.63%) | 7(18.42%) | 10(90.91%) | 23(28.75%) | 15(44.12%) | 23(76.67%) | 2(5.26%) | 7(17.95%) | 2(15.38%) |

**Supplementary Table 6** Antifungal resistance rates of major Candida species stratified by TBSA

|  | *Candida albicans* | | | *Candida tropicalis* | | |
| --- | --- | --- | --- | --- | --- | --- |
| Antimicrobial resistance | TBSA<10%  n=10 | TBSA10-49%  n=6 | TBSA>=50%  n=16 | TBSA<10%  n=7 | TBSA10-49%  n=10 | TBSA>=50%  n=29 |
| Voriconazole | 3(30.00%) | 1(16.67%) | 0(0.00%) | 2(28.57%) | 2(20.00%) | 14(48.28%) |
| Fluconazole | 3(30.00%) | 3(50.00%) | 0(0.00%) | 1(14.29%) | 2(20.00%) | 14(48.28%) |

**Supplementary Table 7.** Specimen-type comparison of t-NGS and conventional culture positivity rates

| **Specimen type** | **n** | **t-NGS positive** | **Culture positive** | **C+/N+** | **C+/N-** | **C-/N+** | **C-/N-** | **Discordant pairs** | **Exact McNemar *P*** |
| --- | --- | --- | --- | --- | --- | --- | --- | --- | --- |
| Sputum | 7 | 7/7 (100.0%) | 7/7 (100.0%) | 7 | 0 | 0 | 0 | 0 | 1.0000 |
| Tissue | 5 | 4/5 (80.0%) | 4/5 (80.0%) | 4 | 0 | 0 | 1 | 0 | 1.0000 |
| Blood | 18 | 10/18 (55.6%) | 3/18 (16.7%) | 2 | 1 | 8 | 7 | 9 | 0.0391 |
| BALF | 6 | 6/6 (100.0%) | 5/6 (83.3%) | 5 | 0 | 1 | 0 | 1 | 1.0000 |
| Wound secretion | 4 | 4/4 (100.0%) | 3/4 (75.0%) | 3 | 0 | 1 | 0 | 1 | 1.0000 |
| Overall | 40 | 31/40 (77.5%) | 22/40 (55.0%) | 21 | 1 | 10 | 8 | 11 | 0.0117 |

Notes: C, conventional culture; N, t-NGS; +, positive; -, negative; BALF, bronchoalveolar lavage fluid. Exact McNemar tests were used for paired comparisons because subgroup discordant-pair counts were small. Only discordant pairs (C+/N- and C-/N+) contribute to the McNemar test statistic.
