# Supplementary material for: Epidemiology, pathogen spectrum, and antimicrobial resistance of infections in burn patients stratified by total body surface area: a bicenter study with evaluation of targeted next-generation sequencing
Source: Front Microbiol. 2026 Jul 15;17:1866146. doi: 10.3389/fmicb.2026.1866146 (PMC13415348; doi:10.3389/fmicb.2026.1866146)
Supplement: Supplementary file 6 [file Table_2.DOCX]

###### Supplementary Figure Legends

**Suppl. Figure 1. Distribution of burn etiologies by age and gender**

(A) Distribution of burn causes across different age groups.

(B) Distribution of burn causes according to gender.

Burn etiologies were classified based on clinical records, including flame, scald, electrical, chemical, and contact burns. Data are presented as percentages.

**Suppl. Figure 2. Anatomical distribution of burn sites stratified by total body surface area**

(A) Percentage distribution of anatomical burn sites among patients in different TBSA groups.

(B) Proportional distribution of burn sites across TBSA categories.

Burn sites were categorized according to standard anatomical regions. Data are presented as percentages.

*TBSA： total body surface area.*

**Suppl. Figure 3**. Hospitalization burden of burn patients across TBSA categories

(A) Comparison of total hospitalization costs among burn patients stratified by TBSA.

(B) Length of hospital stay (LOS) across different TBSA groups.

Hospitalization cost data are presented as median with interquartile range. LOS is expressed in days. Comparisons among groups were performed using the Kruskal–Wallis test.

*TBSA: total body surface area; LOS: length of stay.*

**Suppl. Figure 4. Distribution of detected pathogens in positive specimens**

Sample count distribution of each detected pathogen in 32 positive specimens. The number of samples corresponding to each pathogen (identified via culture- and t-NGS-based methods) among 32 positive specimens is presented in this bar chart, where the count indicates the number of specimens with successful pathogen identification.

###### Supplementary Tables Legends

Suppl. Table 1. Frequency/prevalence of complications among infected burn patients

Frequency of major complications among infected burn patients categorized by TBSA (< 10%, 10–49%, and ≥ 50%). Major complications and infection-related clinical events are listed by TBSA category.

Data are presented as number and percentage. Intergroup comparisons were performed using the chi-square test.

TBSA, total body surface area; MODS, multiple organ dysfunction syndrome; ARDS, acute respiratory distress syndrome; PTSD, post-traumatic stress disorder.

*TBSA, total body surface area; MODS, multiple organ dysfunction syndrome; ARDS, acute respiratory distress syndrome; PTSD, post-traumatic stress disorder.*

Suppl. Table 2. Clinical characteristics of infected burn patients stratified by total body surface area.

Baseline clinical characteristics, burn etiologies, and clinical management parameters of infected burn patients categorized by TBSA.

Data are presented as number and percentage. Intergroup comparisons were performed using the chi-square test.

Suppl. Table 3**. Distribution of bacterial and fungal isolates among burn patients stratified by TBSA**

Counts and percentages for Gram-positive (e.g., Staphylococcus aureus: 22.17% mild to 1.69% severe), Gram-negative (e.g., Klebsiella pneumoniae: 2.37% to 18.92%), and fungi (e.g., Candida tropicalis: 1.18% to 5.69%) across 1656 isolates. Chi-square or Fisher's exact tests; P<0.001 for trends.

Suppl. Table 4**. Antimicrobial resistance rates of major Gram-negative bacteria stratified by TBSA**

Percentages for major Gram-negative bacteria (e.g., Acinetobacter baumannii: ceftazidime 40% mild to 91.30% severe), showing increasing resistance with TBSA. Tested via automated systems; n per group in headers; trends via chi-square (P<0.001)

.

Suppl. Table 5**. Antimicrobial resistance rates of major Gram-positive bacteria stratified by TBSA**

Resistance rates of major Gram-positive bacteria to antimicrobials stratified by TBSA. Percentages for species (e.g., Staphylococcus aureus: oxacillin 21.37% mild to 72.73% severe), with upward trends; no vancomycin resistance. Tested via automated systems; n per group in headers; trends via chi-square (P<0.001).

Suppl. Table 6**. Antifungal resistance rates of major Candida species stratified by TBSA**

Resistance rates of major fungi to antimicrobials stratified by TBSA. Percentages for Candida albicans (voriconazole: 30% mild to 0% severe) and C. tropicalis (48.28% severe), showing species-specific trends. Automated testing; n in headers.

Suppl. Table 7. Specimen-type comparison of t-NGS and conventional culture positivity rates

Paired culture and t-NGS results were compared within each specimen type using exact McNemar tests because discordant-pair counts were small. The table reports n, positivity rates, paired 2 x 2 counts, discordant-pair counts, and exact P values.
